# Reporter gene expression reveals precise auxin synthesis sites during fruit and root development in wild strawberry

**DOI:** 10.1093/jxb/ery384

**Published:** 2018-10-27

**Authors:** Jia Feng, Cheng Dai, Huifeng Luo, Yafan Han, Zhongchi Liu, Chunying Kang

**Affiliations:** 1 Key Laboratory of Horticultural Plant Biology (Ministry of Education), College of Horticulture and Forestry Sciences, Huazhong Agricultural University, Wuhan, China; 2 National Key Laboratory of Crop Genetic Improvement, College of Plant Science and Technology, Huazhong Agricultural University, Wuhan, China; 3 Department of Cell Biology and Molecular Genetics, University of Maryland, College Park, MD, USA

**Keywords:** Auxin biosynthesis, CRISPR/Cas9, *DR5ver2::GUS*, *Fragaria vesca*, fruit, root, spatial–temporal expression, strawberry, *TARs*, *YUC*s

## Abstract

The critical role of auxin in strawberry fruit set and receptacle enlargement was demonstrated previously. While fertilization is known to trigger auxin biosynthesis, the specific tissue source of fertilization-induced auxin is not well understood. Here, the auxin reporter *DR5ver2::GUS* was introduced into wild strawberry (*Fragaria vesca*) to reveal auxin distribution in the seed and fruit receptacle pre- and post-fertilization as well as in the root. In addition, the expression of *TAR* and *YUCCA* genes coding for enzymes catalysing the two-step auxin biosynthesis pathway was investigated using their respective promoters fused to the β-glucuronidase (GUS) reporter. Two *FveTARs* and four *FveYUCs* were shown to be expressed primarily in the endosperm and embryo inside the achenes as well as in root tips and lateral root primordia. Expression of these reporters in dissected tissues provided more detailed and precise spatial (cell and tissue) and temporal (pre- and post-fertilization) information on where auxin is synthesized and accumulates than previous studies in strawberry. Moreover, we generated CRISPR-mediated knock-out mutants of *FveYUC10*, the most abundant *YUC* in seeds; the mutants had a lower free auxin level in young fruit, but displayed no obvious morphological phenotypes. However, overexpression of *FveYUC10* resulted in elongated hypocotyls in Arabidopsis caused by elevated auxin level. Overall, the study revealed auxin accumulation in the chalazal seed coat, embryo, receptacle vasculature, root tip, and lateral root primordia and highlighted the endosperm as the main auxin biosynthesis site for fruit set.

## Teaser

Auxin distribution revealed by *DR5ver2::GUS* and expression patterns of *FveTARs* and *FveYUCs* determined by the GUS reporter highlighted the endosperm as the main auxin biosynthesis site in strawberry achenes.

## Introduction

Cultivated strawberry (*Fragaria ananassa*) is one of the major fruit crops grown worldwide. Developmentally, strawberry fruit is unique in that the juicy flesh is developed from the receptacle, an enlarged stem tip, while the true fruit, called an achene, is developed from a pollinated ovary containing a single ovule (Darrow, 1966; Hollender *et al.*, 2012). Because of the accessibility of the achenes on the fruit surface, strawberry fruit has long been used as a model system to study the roles of auxin in fruit development. When the achenes are removed after pollination, receptacle fruit fails to develop, but exogenous auxin application can substitute for the achene and restore the fruit development (Nitsch, 1950), suggesting the achene as the source of the auxin for promotion of receptacle fruit set. This early observation was supported later by RNA-seq data and measurement of free auxin. RNA-seq data of carefully staged achenes showed rapid up-regulation of auxin biosynthetic genes in the achenes soon after fertilization (Kang *et al.*, 2013). Measurement of free auxin in the achene showed a dramatic increase starting from 3 d after fertilization and reaching a maximum of ~70 ng g^−1^ fresh weight at about 12 d after fertilization (Nitsch, 1950, 1955; Dreher and Poovaiah, 1982; Symons *et al.*, 2012). By contrast, auxin biosynthesis genes were not expressed in the receptacle during early-stage fruit development (Kang *et al.*, 2013) and free auxin in the fruit receptacle was about 5 ng g^−1^ fresh weight, significantly lower than that in the achenes (Dreher and Poovaiah, 1982; Symons *et al.*, 2012; Estrada-Johnson *et al.*, 2017). Together, these observations suggest that fertilization-induced auxin biosynthesis primarily occurs in the achenes of strawberry.

Most of our basic understanding of auxin synthesis and signaling was derived from molecular genetic studies in Arabidopsis, including the revelation of the two-step biosynthetic pathway of indole-3-acetic acid (IAA), the major auxin. The tryptophan aminotransferase of Arabidopsis 1 (TAA1)/TAR family of aminotransferases convert tryptophan to indole-3-pyruvic acid (IPyA), and the YUCCA (YUC) family of flavin-containing monooxygenases convert IPyA to IAA (Mashiguchi *et al.*, 2011; Stepanova *et al.*, 2011; Won *et al.*, 2011). Multiple *TARs* and *YUCs* in Arabidopsis play similar roles in embryogenesis, vascular patterning, flower development, and shade avoidance (Zhao *et al.*, 2001; Cheng *et al*., 2006, 2007; Stepanova *et al.*, 2008; Tao *et al.*, 2008; Yamada *et al.*, 2009). The homologs of these *TAA*/*TAR* and *YUC* genes were identified in strawberry (Liu *et al.*, 2012, 2014; Kang *et al.*, 2013) and functional studies were previously conducted for *FaYUC1* and *FaYUC2* in cultivated strawberries and *FvYUC6* in the wild strawberry, indicating roles in leaf, root, flower, and fruit development (Liu *et al.*, 2012, 2014). During late-stage fruit development (green stage to ripe), *FaYUC2*, *FaTAR2*, and *FaTAA1* were shown to be expressed in the receptacle fruit, and transient silencing of *FaTAR2* in ripening fruit resulted in altered auxin response (Liu *et al.*, 2014; Estrada-Johnson *et al.*, 2017). These studies suggest that certain levels of auxin biosynthesis in the receptacle may contribute to fruit ripening, which is unexpected given the non-climacteric fruit ripening of strawberry and that auxin was previously thought of as an inhibitor of fruit ripening (Given *et al.*, 1988; Chai *et al.*, 2011; Jia *et al.*, 2011; Li *et al.*, 2011).

Here, we focus on understanding the role of auxin in early fruit development, specifically fruit set, taking advantage of existing transcriptome data of wild strawberry (*Fragaria vesca*) fruit tissues (Kang *et al.*, 2013). The comprehensive dataset included five finely dissected fruit tissue types at five early fruit developmental stages. Specifically, the achene was dissected into ovary wall, ghost (seed coat and endosperm), and embryo; the receptacle was dissected into pith and cortex. *FveTARs* and *FveYUCs* were found more abundantly expressed in the ghost than in the embryo, suggesting that auxin biosynthesis occurred mainly in the ghost. However, exactly where auxin is produced inside the achene (seed coat or endosperm, or specific regions of the seedcoat or endosperm) is not known. Given the multiple family members in the genome, understanding which *FveTARs* and *FveYUCs* are responsible for the fertilization-induced auxin production within the achene or specific tissues of the root will be necessary. Local auxin production is highly relevant to auxin functions (Robert *et al.*, 2013; Chen *et al.*, 2014). Identification of the tissue and timing of auxin biosynthesis will provide crucial insights into auxin-mediated developmental processes and aid in future manipulation of auxin for increasing fruit yield.

Our study was conducted in wild strawberry, an emerging model for the cultivated strawberry due to its diploid genome and abundant genomic and molecular resources (Slovin *et al.*, 2009; Kang *et al.*, 2013; Edger *et al.*, 2018). We first utilized an auxin-responsive synthetic promoter, *DR5ver2* (Liao *et al.*, 2015), to visualize dynamic auxin distributions in the *F. vesca* fruit before and after fertilization and in the *F. vesca* root. *DR5ver2* is a new and improved synthetic promoter containing nine copies of the Aux *cis*-element fused to the β-glucuronidase (GUS) reporter. This new *DR5ver2* reporter was shown to be more sensitive to auxin than the earlier version, *DR5* (Liao *et al.*, 2015). In addition, we generated transgenic *F. vesca* plants containing *promoter::GUS* reporters of two *FveTAR* and four *FveYUC* genes. Analyses of these reporter gene expression patterns revealed distinct spatial and temporal patterns, suggesting the unique roles each gene may play during fruit and root development. To investigate the biological significance of auxin in strawberry fruit development, CRISPR/Cas9 was used to knock out *FveYUC10*. The resulting *fveyuc10* mutants did not exhibit obvious morphological defects, but showed a significant reduction of free auxin. Taken together, this work revealed the sites of auxin biosynthesis within achenes and the major auxin biosynthesis genes responsible in fruit and root with a resolution at the tissue and cell level; it indicates the endosperm is an important tissue for fertilization-induced auxin biosynthesis.

## Materials and methods

### Plant materials and growth conditions

*Fragaria vesca* strain Hawaii 4 (PI551572, National Clonal Germplasm Repository, USA, white-fruited) was used for transformation and served as the wild type control for qRT-PCR. The Arabidopsis Columbia accession was used as the wild type. These strawberry and Arabidopsis plants were cultivated in a growth room under a light intensity of 100 μmol m^−2^ s^−1^ with a 16/8 h light/dark photoperiod at 22 °C.

### Plasmid construction

For *DR5ver2::GUS*, *DR5ver2* with nine repeats of a higher affinity auxin response element (AuxREs, TGTCGG) was synthesized as previously described (Liao *et al.*, 2015) and inserted into the binary vector pMDC162 (Curtis and Grossniklaus, 2003) before the *GUS* gene by gateway cloning (Invitrogen). For promoter::GUS constructs, the following promoters were used: 2005 bp promoter of *FveTAR1* (gene37056 (ver2.0.a2); gene31791 (ver1.1); FvH4_5g05900 (ver4.0)), *FveTAR2* (gene31790/FvH4_5g05880, 1907 bp), *FveYUC4* (gene11728/FvH4_2g29930, 1958 bp), *FveYUC5* (gene32686/FvH4_2g14550.1, 1971 bp), *FveYUC10* (gene27796/FvH4_2g24750, 1411 bp), and *FveYUC11* (gene06886/FvH4_4g17980, 2000 bp). The promoter fragments were amplified from the genomic DNA of YW5AF7, a seventh generation inbred line of *F. vesca* (Slovin *et al.*, 2009), and inserted into the binary vector pHGWFS7 before the *GFP-GUS* dual reporter by gateway cloning. To construct the single guide RNA (sgRNA)-Cas9 vector for *FveYUC10*, two sgRNAs respectively targeting *FveYUC10* at 8 bp (sgRNA1: AAGCGGCGGTGATAATAGT) and 728 bp (sgRNA2: ATGGAGACCTGGCCAAGTA) downstream of the translation initiation codon were designed using the web server CRISPR-P (http://cbi.hzau.edu.cn/cgi-bin/CRISPR). Two *AtU6 promoter-sgRNA-AtU6 terminator* cassettes were amplified by PCR using *pCBC-DT1T2* as the template, and then the PCR fragments were inserted into pHSE401 (Xing *et al.*, 2014) by Golden Gate Assembly and confirmed by Sanger sequencing. For overexpressing *FveYUC10*, the coding sequence was PCR amplified and cloned into the *Eco*RI- and *Nde*I-digested binary vector pRI101 to fuse with green fluorescent protein (GFP) by Gibson assembly. These constructs were transformed into *Agrobacterium tumefaciens* strain GV3101 for plant transformation. The primers used for making these constructs are shown in Supplementary Table S1 at *JXB* online.

### Strawberry transformation

Strawberry transformation was carried out as previously described with minor modifications (Kang *et al.*, 2013). Briefly, callus was induced on 5^++^ medium (1×Murashige and Skoog (MS), 2% sucrose, 3.4 mg l^−1^ benzyladenine, 0.3 mg l^−1^ indole-3-butyric acid (IBA), and 0.7% phytoagar, pH 5.8) from the leaf strips of Hawaii 4 for 2 weeks in the dark; co-cultivated with *Agrobacterium* GV3101 harboring each construct for 1 h in co-cultivation buffer (1×MS, 2% sucrose, 0.4 mg l^−1^ acetosyringone) with gentle shaking; and then transferred to the 5^++^ medium, and kept for 3 d in the dark. The leaf strips were washed with sterile water to remove *Agrobacterium* and transferred to 5^++^ medium with 250 mg l^−1^ timentin and 250 mg l^−1^ carbnicillin for 2 weeks in the dark. The calli were transferred to new medium every 14 d on the 5^++^ medium with 250 mg l^−1^ timentin, 250 mg l^−1^ carbnicillin and up to 4 mg l^−1^ hygromycin until shoots appeared. The shoots were transferred to the rooting medium (0.5×MS, 0.01 mg l^−1^ IBA, 2% glucose, 250 mg l^−1^ timentin, 250 mg l^−1^ carbnicillin, 4 mg l^−1^ hygromycin, and 0.7% phytoagar, pH5.8). After 1–2 months, the regenerated plants were transferred to the soil and cultivated in the growth room for future analysis. The transformation efficiency (based on number of viable shoots among the total number of leaf strips) is about 5%.

### Stable transformation in Arabidopsis

Arabidopsis transformation was carried out by the floral-dip method (Clough and Bent, 1998). T1 transgenic seeds were screened on half-strength MS medium (M5524, Sigma-Aldrich) with 100 mg l^−1^ kanamycin. T2 plants were used for characterization.

### GUS staining

The ovules or seeds were dissected out of the achenes under a stereomicroscope from T0 transgenic plants. More than 10 samples per tissue and developmental stage were examined. Representative images are shown in the figures. Receptacles and roots were also collected from T0 plants. The tissues were stored in cold 90% acetone during dissection and then kept at room temperature for 20 min. Next, acetone was removed and GUS staining solution (1 mM X-glucuronic acid, 0.1 mM EDTA, 0.1% Triton X-100, and 10 mM (for seed) or 2mM (for root) potassium ferri/ferrocyanide in 100 mM phosphate buffer, pH 7.0) was added to submerge all the material. After 30 min of vacuum, tissues were incubated overnight at 37 °C. The samples were then mounted on clearing solution (chloral hydrate: glycerol: H_2_O: 8:1:1) for 5 h and observed under differential interference contrast (DIC) optics using a Zeiss Axioscope A1 microscope with a ×0.5 optical adapter. The images were captured, analysed, and exported using ZEN2.3.

### RNA extraction and qRT-PCR

Total RNA was extracted using a Plant Total RNA Isolation Kit (Sangon Biotech, Shanghai, China, no. SK8631) following the manufacturer’s instructions. RNA quality was examined by both gel electrophoresis for sharp rRNA bands and the NanoDrop 2000 Spectrophotometer to reach an OD_260/280_ of 1.8. Approximately 1 μg of total RNA was used for cDNA synthesis using a PrimeScript^TM^ RT reagent kit (TaKaRa, Shiga, Japan, cat. no. RR047A), which removes genomic contamination with gDNA eraser digestion before reverse transcription. A total volume of 10 μl qPCR reaction mixture contained 5 μl of 2×SYBR Green PCR master mix (cat. no. 172-5124, Bio-Rad), 1 μl of 5× diluted cDNA, 0.25 μl of each primer (Supplementary Table S1), and 3.5 μl ddH_2_O. Amplification was performed using a QuantStudio 7 Flex system (Applied Biosystems, USA). The amplification program consisted of one cycle of 50 °C for 2 min and 95 °C for 10 min, followed by 50 cycles of 95 °C for 15 s, 60 °C for 20 s, and 72 °C for 20 s. The fluorescent product was detected at the third step of each cycle. The expression level of each gene was calculated using the $2^{-\Delta \Delta C_{T}}$ method (Livak and Schmittgen, 2001). Primers were designed to span exon to exon junctions if allowed. *Gene11892* and *actin* (At3g18780) were used as the control genes. Three biological replicates were used.

### Phylogenetic analysis

The protein sequences were obtained from the *F. vesca* genome annotation ver2.0.a2 (Li *et al.*, 2018) or downloaded from The Arabidopsis Information Resource (TAIR) (http://www.arabidopsis.org/). The sequence alignment was performed using Clustal Omega (http://www.ebi.ac.uk/Tools/msa/clustalo). An unrooted phylogenetic tree was constructed using MEGA 7 (http://www.megasoftware.net/) with the neighbor-joining statistical method and bootstrap analysis (1000 replicates).

### Heatmap

Sequence read counts of the *FveTARs* and *FveYUCs* were quantified with the reads per kilobase per million mapped reads (RPKM) unit based on the *F. vesca* RNA-seq dataset (Li *et al.*, 2018). A heatmap was made by using MultiExperiment Viewer 4.8 (MeV4.8; http://www.tm4.org/mev/). In the heatmap, the average RPKM value among the replicates was used to represent the expression pattern of each gene (Supplementary Table S2).

### Hormone treatment

Newly opened flowers were emasculated to prevent self-fertilization and then sprayed with 500 μM 1-naphthylacetic acid (NAA; Sigma-Aldrich) every 8 h. The ovules and fruit receptacles were collected for GUS staining at 2 and 5 d post-treatment, respectively. Roots obtained from adult plants grown in the substrate were immersed in 100 μM NAA for 3 h and then subjected to GUS staining.

### Auxin quantification

Entire fruit (achenes plus receptacle) at stage 4 were collected, weighed, and then immediately frozen in liquid N_2_. The wild type and *fveyuc10* (L2 and L3 pooled together) samples with four biological replicates were analysed by liquid chromatography–electrospray ionization–quadrupole time-of-flight tandem mass spectrometry as previously described (Ng *et al.*, 2015).

### Statistical analyses

Statistical analyses were performed with SPSS v22.0 (IBM Corp., Armonk, NY, USA). Pairwise comparisons were determined using Student’s *t*-test (**P<0.01). The comparison between multiple samples was determined using Tukey’s test, and significant differences at the *P*<0.05 level are indicated by different letters.

### Accession numbers

Gene accessions for *FveTARs* and *FveYUCs* in the ver2.0.a2 annotation (Li *et al.*, 2018) and the ver4.0 annotation (Edger *et al.*, 2018) are as follows: gene03586/FvH4_4g25850 (*FveTAA1*), gene37056/FvH4_5g05900 (*FveTAR1*), gene31790/FvH4_5g05880 (*FveTAR2*), gene14327/FvH4_ 7g02760(*FveTAR3*), gene27578/FvH4_2g13010 (*FveTAR4*), gene01005/FvH4_2g15120.1 (*FveYUC1*), gene30882/FvH4_1g04800 (*FveYUC2*), gene08377/FvH4_2g20150.1 (*FveYUC3*), gene11728/FvH4_2g29930 (*FveYUC4*), gene32686/FvH4_2g14550.1 (*FveYUC5*), gene08779/FvH4_2g36220.1 (*FveYUC6*), gene00528/FvH4_4g05280 (*FveYUC7*), gene27796/FvH4_2g24750 (*FveYUC10*), and gene06886/FvH4_4g17980 (*FveYUC11*). Gene accessions for *AtTARs* and *AtYUCs* in Arabidopsis are as follows: At1g70560 (*AtTAA1*), At1g23320 (*AtTAR1*), At4g24670 (*AtTAR2*), At1g34040 (*AtTAR3*), At1g34060 (*AtTAR4*), At4g32540 (*AtYUC1*), At4g13260 (*AtYUC2*), At1g04610 (*AtYUC3*), At5g11320 (*AtYUC4*), At5g43890 (*AtYUC5*), At5g25620 (*AtYUC6*), At2g33230 (*AtYUC7*), At4g28720 (*AtYUC8*), At1g04180 (*AtYUC9*), At1g48910 (*AtYUC10*), and At1g21430 (*AtYUC11*).

## Results

### Auxin distribution and dynamics in the achene and receptacle fruit of *F. vesca*

**Fig. 1.**
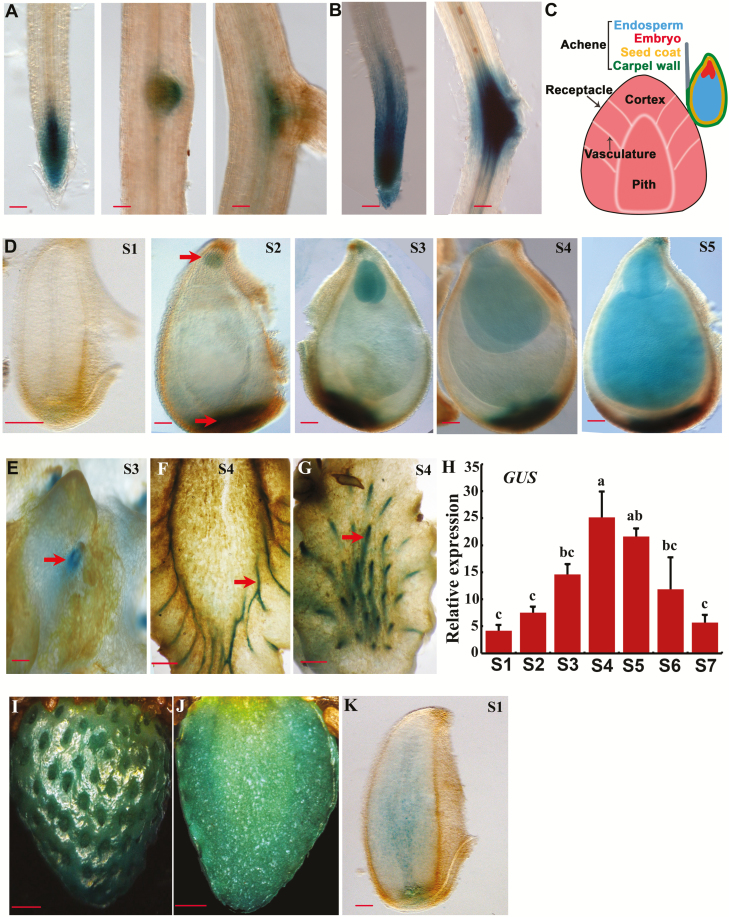
*DR5ver2::GUS* expression in roots and fruit of *F. vesca*. (A) *GUS* expression of the *DR5ver2::GUS* reporter lines in root. (B) *GUS* expression in root after NAA (100 μM) treatment. (C) Diagram of strawberry fruit showing the receptacle and an achene. The achene consists of embryo, ghost (endosperm and seed coat), and the ovary wall. (D) *GUS* expression of *DR5ver2::GUS* in ovule (stage 1) and seeds at stages 2–5. Arrows indicate embryo (top) and chalazal seed coat (bottom). (E–G) *GUS* expression of *DR5ver2::GUS* in receptacle. (E) Surface of the receptacle at stage 3. Arrow indicates the region where achene is attached to the receptacle. (F, G) Different sections of the receptacle at stage 4. Arrows indicate the vasculatures in sectioned receptacle fruit. (H) Expression level of *GUS* obtained by qRT-PCR in the receptacle of the *DR5ver2::GUS* reporter line at stages 1–7. *Gene11892* was used as the internal control. Data are means ±SD obtained from three biological replicates. Significant differences at the *P*<0.05 level are indicated by different letters, tested by Tukey’s test. (I–K) GUS staining in receptacle (I, J) and ovule (K) at 5 and 2 d post-NAA (500 μM) treatment. Scale bars: 10 µm (A, B, D, K); 100 µm (E); 2 mm (F, G, I, J).

As strawberry is a unique fruit, revealing auxin distribution in its achenes and receptacle during early-stage fruit development would help understand the developmental process underlying fruit set. To do this, *DR5ver2* was used to drive the *GUS* gene, and the reporter construct was stably transformed into the *F. vesca* accession Hawaii 4. A total of 12 independent transgenic lines were obtained and validated by PCR amplification of the *DR5ver2* fragment (Supplementary Fig. S1A). As auxin distribution in roots was well documented in Arabidopsis, GUS staining was first carried out in *F. vesca* roots to assess whether *DR5ver2::GUS* acts correctly in these *F. vesca* transgenic lines in the T0 generation. *GUS* was expressed in the entire root tip with a higher level in the stele of the root meristematic zone (Fig. 1A). Compared with the expression pattern of *DR5::GUS* in Arabidopsis and garden strawberry (Ulmasov *et al.*, 1997; Ottenschläger *et al.*, 2003; Estrada-Johnson *et al.*, 2017), *GUS* was more highly expressed, probably owing to the use of an enhanced *DR5* (Liao *et al.*, 2015). In addition, *DR5ver2::GUS* was expressed at the tip of a lateral root primordium before emergence and at the base of an emerged young lateral root (Fig. 1A). Moreover, GUS staining became stronger both at the root tip and at the lateral root primordium in response to NAA treatment (Fig. 1B), suggesting that the *DR5ver2::GUS* reporter behaves properly and can be used to monitor auxin distribution in *F. vesca*.

In strawberry, the achenes, i.e. the true fruit, sit on the surface of the receptacle (Fig. 1C); each achene consists of the ovary wall encasing a single seed. Each seed consists of seedcoat, endosperm, and embryo (Fig. 1C). The developmental stages of early fruit development were previously defined (Hollender *et al.*, 2012; Kang *et al.*, 2013) with embryo morphologies as temporal markers. Specifically, stage 1 is defined as flower anthesis, a pre-fertilization stage; stage 2 is only 2–3 d post-fertilization with a globular shaped embryo; stage 3 is defined as having fruits with a heart-shaped embryo; stage 4 fruit has a torpedo-shaped embryo; stage 5 has a fully grown embryo. The *DR5ver2::GUS F. vesca* transgenic lines were first examined in ovules (stage 1) and seeds at stages 2–5. There was no staining in ovules at anthesis (stage 1), indicating a very low level of free auxin (Fig. 1D). Starting from stage 2, which correlates post-fertilization, GUS was observed in the chalazal seed coat and embryo (Fig. 1D). *GUS* expression was increased from stage 2 to stage 5 in embryo and chalazal seed coat but less so in the endosperm (Fig. 1D).

Previous work suggested that post-fertilization-induced auxin in the achenes was transported to the receptacle to stimulate fleshy fruit growth (Nitch 1950; Kang *et al.*, 2013). To reveal dynamic auxin accumulation in the receptacle, we examined GUS reporter expression in stage 3 and 4 receptacles of the *DR5ver2::GUS* transgenic lines. Interestingly, *GUS* was specifically expressed in the receptacle vasculature strands that connect to the achene, consistent with previous work (Estrada-Johnson, *et al.*, 2017) and suggesting a likely auxin transport route from the chalazal seed coat to the vasculature veins of the receptacle (Fig. 1E, F, G). To quantitatively determine the auxin reporter expression, qRT-PCR was used to examine *GUS* transcripts driven by *DR5ver2* in fruit receptacles at stages 1–5 plus later stages (stages 6 and 7). Stage 6 is equivalent to the white stage, while stage 7 is equivalent to the ripe/red stage. The *GUS* transcript level gradually increased from stage 1 to stage 4 and then slowly reduced from stage 4 to stage 7 (Fig. 1H). The stage 1–4 expression trend positively correlates with the early-stage receptacle fruit enlargement. Moreover, GUS expression level was greatly enhanced in the developing receptacle as well as ovule at anthesis in response to NAA treatment (Fig. 1I, J, K). Collectively, auxin distribution in seeds, roots, and fruit receptacles was revealed by the *DR5ver2::GUS* reporter gene expression in transgenic *F. vesca* plants, which not only corroborates prior studies suggestive of fertilization-induced auxin synthesis and transport from achene to the receptacle, but also reveals the likely transport route from chalazal seed coat to the receptacle via the vasculature strands connecting the two.

### Phylogenetic and expression analyses of *FveTARs* and *FveYUCs*

**Fig. 2.**
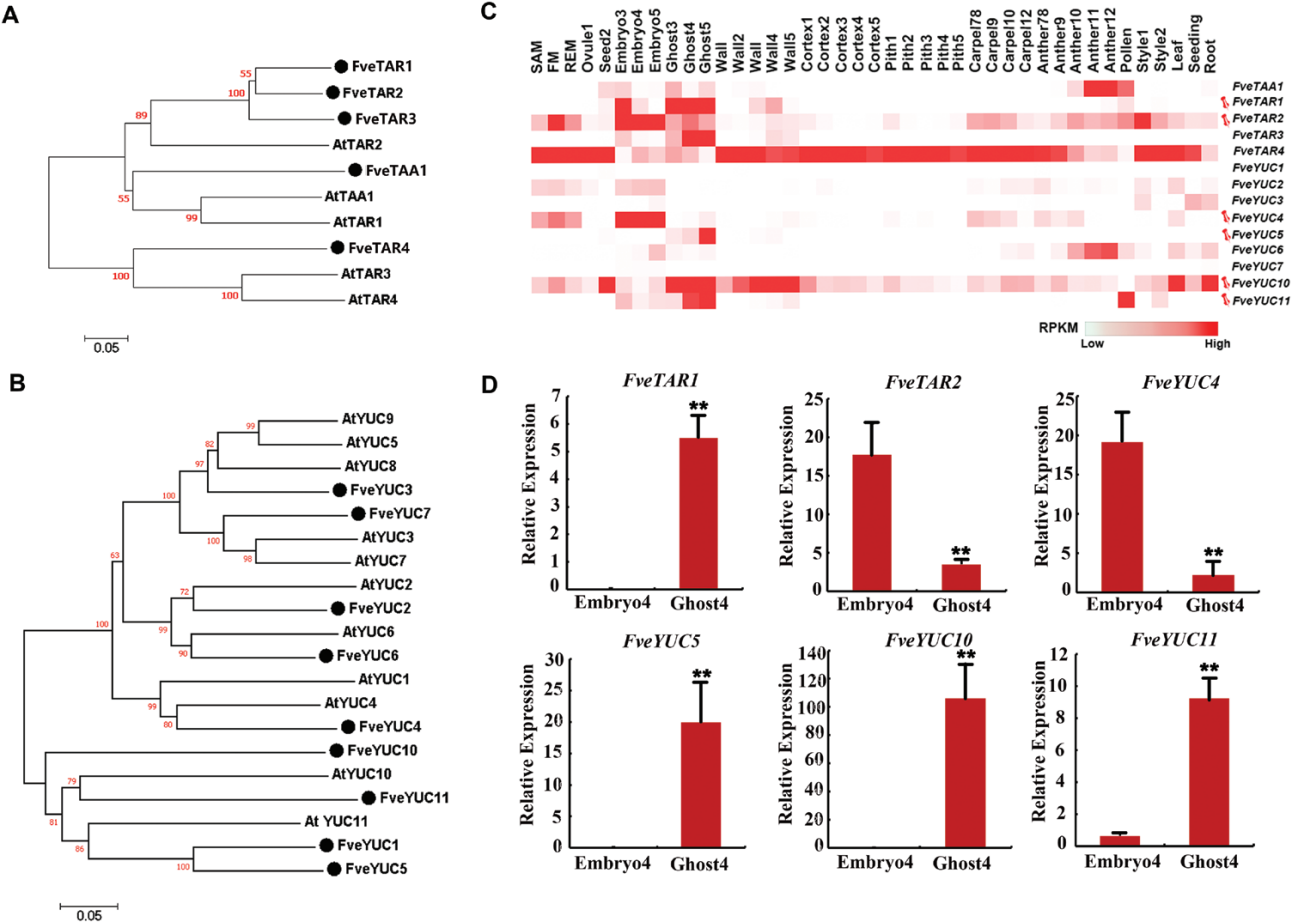
Phylogenetic analysis and expression pattern of *FveTAR*s and *FveYUC*s in *F. vesca*. (A, B) The phylogenetic tree of *TARs* (A) and *YUCs* (B) in *F. vesca* and Arabidopsis. (C) Heatmap showing expression patterns of *FveTARs* and *FveYUCs* represented by normalized RPKM derived from the RNA-seq dataset (Li *et al.*, 2018). Six genes selected for promoter activity analysis are marked by pins. (D) Expression level of the six selected genes obtained by qRT-PCR in embryo and ghost at stage 4. *Gene11892* was used as the internal control. Data are means ±SD obtained from three biological replicates. **P<0.01, Student’s *t*-test.

*TARs* and *YUCs* encode the two enzymes catalysing the main auxin biosynthetic pathway (Mashiguchi *et al.*, 2011; Stepanova *et al.*, 2011; Won *et al.*, 2011). Although the phylogenetic analyses of these genes have been performed in both wild and cultivated strawberry (Kang *et al.*, 2013; Liu *et al.*, 2014; Estrada-Johnson *et al.*, 2017), the recently published high quality genome and annotation of *F. vesca* (Edger *et al.*, 2018; Li *et al.*, 2018) necessitates an updated phylogenetic tree (Fig. 2A, B). Gene expression data of *FveTARs* and *FveYUCs* are included in the same figure to facilitate comparisons of gene expression patterns among family members (Fig. 2). Specifically, five *FveTARs* (*FveTAA1* and *FveTAR1–4*) and nine *FveYUCs* (*FveYUC1–7* and *10–11*) were identified in *F. vesca*. *FveTAR1*, *FveTAR2*, and *FveTAR3* shared a high level of similarity to *AtTAR2*; *FveTAA1* was grouped with *AtTAA1* and *AtTAR1*; *FveTAR4* was grouped with *AtTAR3* and *AtTAR4* (Fig. 2A). *FveYUCs* were divided into two large groups. The first group contained *FveYUC1*, *5*, *10*, and *11*, which were clustered with *AtYUC10* and *AtYUC11*, while the second group contained the other five *FveYUCs* (*2*, *3*, *4*, *6*, and *7*) (Fig. 2B).

The RNA expression of each of the *FveTARs* and *FveYUCs* was examined by mining the comprehensive RNA-seq data representing 41 different tissues, mostly floral organs and fruit tissues of *F. vesca* (Kang *et al.*, 2013; Hollender *et al.*, 2014; Toljamo *et al.*, 2016; Li *et al.*, 2018). Different members of *FveTARs* and *FveYUCs* exhibit distinct expression patterns (Fig. 2C; Supplementary Table S2). *FveTAA1* is highly expressed in anthers at late developmental stages and mature pollens. *FveTAR2* is more abundant in embryos. *FveTAR1* and *FveTAR3* are highly expressed in ghosts. *FveTAR4* is highly and broadly expressed. In the *YUC* gene family, *FveYUC1*, *2*, *3*, and *7* are barely expressed. *FveYUC6* is specifically expressed in anthers. *FveYUC4* is more abundant in embryos, while *FveYUC5*, *10*, and *11* are highly expressed in ghosts. Of note, none of the *FveTARs* or *FveYUCs* was significantly expressed in fruit receptacles, including cortex and pith, with the exception of *FveTAR4*, which appears to be a ubiquitously expressed gene. Finally, *FveTAR1*, *FveTAR2*, and four *FveYUCs* (*4*, *5*, *10*, and *11*) were selected to investigate their expression further, as they are likely more relevant to auxin-mediated fruit set due to their abundant and specific expression in the seed. The expression of these six genes was confirmed by qRT-PCR in embryos and ghosts at stage 4, which was consistent with the transcriptome data (Fig. 2C, D).

### Detailed analysis of GUS reporter expression of *FveTAR2* and *FveYUCs* in seeds

**Fig. 3.**
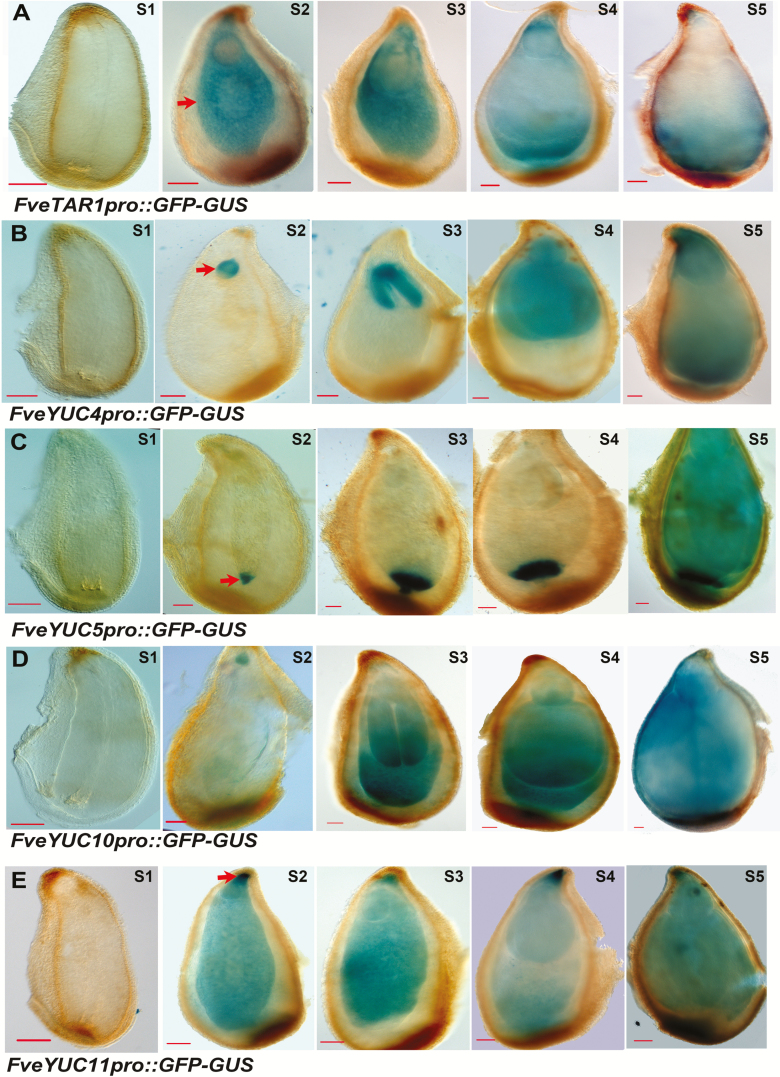
Expression pattern of *FveTARs* and *FveYUCs* in seeds. (A–E) *GUS* expression of the *FveTAR1pro::GFP-GUS* (A), *FveYUC4pro::GFP-GUS* (B), *FveYUC5pro::GFP-GUS* (C), *FveYUC10pro::GFP-GUS* (D), and *FveYUC11pro::GFP-GUS* (E) reporter lines in stage 1 ovule and stages 2–5 seed, respectively. Arrows indicate endosperm (A), embryo (B), chalazal endosperm (C), and the micropylar pole of the seed (E). Scale bars: 10 µm (A–E).

To gain finer tissue resolution of expression for each of the six chosen auxin biosynthetic genes, their promoters (~2 kb upstream of the translational start codon; Supplementary Fig. S2) were respectively isolated and used to drive the *GFP-GUS* dual reporter gene. The constructs were stably transformed into the *F. vesca* variety Hawaii 4. With the exception of *FveTAR2*, fertile transgenic plants for the other five genes were successfully obtained. Two to eight independent transgenic lines for each construct were confirmed by PCR (Supplementary Fig. S1) and then characterized. The ovules and seeds at stages 2–5 of other reporter lines were respectively stained with X-Gluc. For *FveTAR1pro::GFP-GUS*, no blue color was detected in ovules (stage 1, pre-fertilization), indicating low or no expression of *TAR1*; however, at stage 2 (soon after fertilization) to stage 5, the *FveTAR1* reporter was almost exclusively expressed in the endosperms and at a high level (Fig. 3A). None of the four *FveYUCs* showed reporter expression in stage 1 ovules; however, their promoters were all induced to a high level post-fertilization (Fig. 3B–E). This strongly suggests that fertilization plays a key role in inducing the biosynthesis of auxin in the seed. Strikingly, these four *FveYUCs* genes displayed a great divergence in their post-fertilization expression patterns in seeds (Fig. 3B–E). *FveYUC4* was predominantly expressed in the embryos at stages 2–5 (Fig. 3B), indicating that *FveYUC4* may catalyse auxin biosynthesis primarily for regulating embryogenesis. Among the other three *FveYUC* genes, *FveYUC5* was exclusively expressed in the chalazal endosperms from stage 2 to stage 5 (Fig. 3C) and *FveYUC10* and *FveYUC11* were more evenly expressed in the entire endosperms post-fertilization (Fig. 3D, E). *YUC10*’s GUS expression is not as robust as one would expect based on its high RNA-seq reads and qRT-PCR result (Fig. 2C, D). One explanation might be that the 1.4 kb promoter used in the construct lacks some important regulatory elements. *FveYUC11* was also highly expressed at the micropylar pole of the seed next to the base of the embryo (Fig. 3E). Interestingly, no GUS staining was observed in fruit receptacles of all of the *TARs* and *YUCs* reporter lines (Supplementary Fig. S3), suggesting that auxin synthesis may not occur in the receptacle at early-stage fruit development. Taken together, GUS reporter expression revealed specific and diverse expression patterns of different *FveTAR* and *FveYUC* genes within the seeds during early-stage fruit development, highlighting the endosperm as the main site of post-fertilization auxin biosynthesis.

### Expression pattern of *FveTARs* and *FveYUCs* in roots revealed by the GUS reporters

**Fig. 4.**
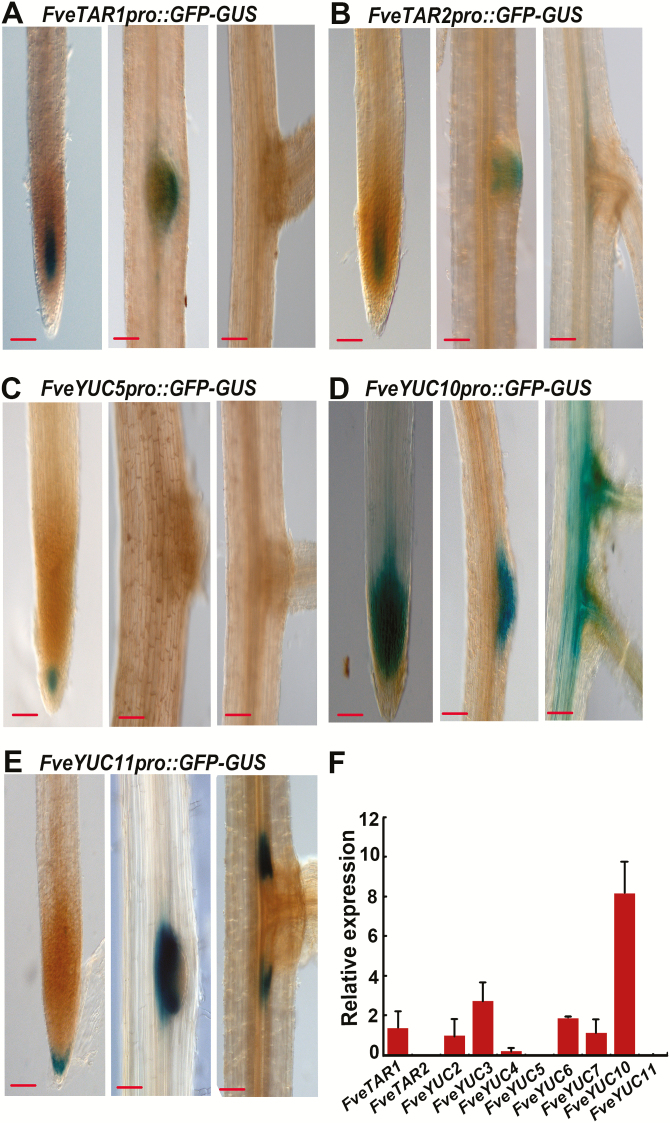
Expression pattern of *FveTARs* and *FveYUCs* in roots. (A–E) *GUS* expression of the *FveTAR1pro::GFP-GUS* (A), *FveTAR2pro::GFP-GUS* (B), *FveYUC5pro::GFP-GUS* (C), *FveYUC10pro::GFP-GUS* (D), and *FveYUC11pro::GFP-GUS* (E) reporter lines in root. Scale bars: 10 µm (A–E). (F) Expression level of *FveTARs* and *FveYUCs* in the roots of wild type obtained by qRT-PCR. *Gene11892* was used as the internal control. Data are means ±SD obtained from three biological replicates.

Onsite auxin biosynthesis is also crucial for root patterning (Benková *et al*., 2003). Hence, we examined the expression pattern in the roots of these reporter lines. Roots of the T0 transgenic plants were collected from soil and stained for GUS. The results showed that *FveTAR1* and *FveTAR2* were similarly expressed in the stele of the meristematic zone and in the emerging lateral root primordia (Fig. 4A, B). No staining was observed in roots of the *FveYUC4pro::GFP-GUS* reporter lines. *FveYUC5* was only expressed around the quiescent center at a low level (Fig. 4C). By contrast, *FveYUC10* was highly expressed in the root meristematic zone, in the entire lateral root primordia, and in the vasculature of subtending roots (Fig. 4D). *FveYUC11* was expressed in the columella cells and at the margins of lateral roots (Fig. 4E). Among the six genes examined, *FveYUC10* was the most abundantly expressed in roots, which was independently confirmed by qRT-PCR (Fig. 4F). The results revealed distinct expression patterns of these auxin biosynthetic genes in roots, demonstrating that the promoters used here possess distinct regulatory elements endowing these genes with specific expression patterns.

### Characterization of the *fveyuc10* mutants created by CRISPR/Cas9 in *F. vesca*

Since *FveYUC10* is most abundantly expressed in seeds and roots among the *FveYUC*s (Figs 2C, D, 4D), we hypothesized that genetic manipulation of *FveYUC10* will likely give rise to severe morphological phenotypes and hence reveal the biological significance of auxin biosynthesis in strawberry fruit development. To test this possibility, *FveYUC10* (Supplementary Fig. S4) driven by the constitutive 35S promoter was first transformed into wild type Arabidopsis ecotype Col-0. Expression of *FveYUC10* was verified by qRT-PCR (Supplementary Fig. S5A). As overexpression of *AtYUCs* usually resulted in elongated hypocotyls (Hentrich *et al.*, 2013; Zhao *et al.*, 2001), the *35S::FveYUC10* transgenic plants in the T2 generation were grown together with the wild type control under white light. Consequently, the average length of *35S::FveYUC10* hypocotyls was 3.94 mm, significantly longer than the control (1.99 mm, *P*<0.01) (Supplementary Fig. S5B, C). This result indicates that *FveYUC10* should be functional in auxin biosynthesis.

**Fig. 5.**
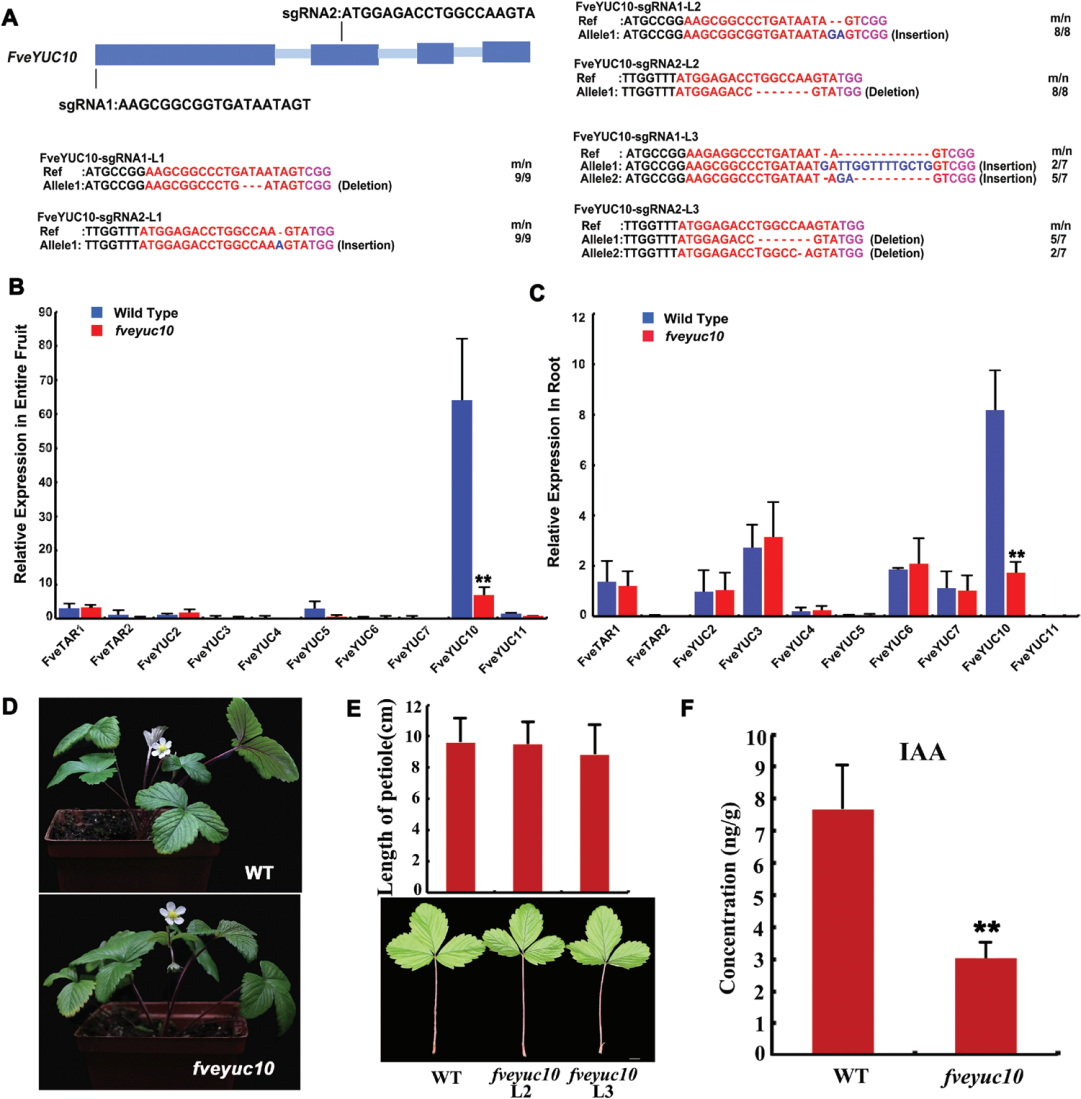
Characterization of the *fveyuc10* mutants created by CRISPR/Cas9. (A) Genotyping of the three *FveYUC10-CRISPR* lines (L1–3) in the T0 generation. The protospacer adjacent motif sequence is indicated with magenta. The sgRNA is indicated with red. The mutation sites are indicated with blue. For the term *m*/*n*, *n* indicates the number of clones examined, and *m* indicates the number of clones showing the indicated genotype. (B) Expression level of *FveTARs* and *FveYUCs* in fruit (stage 4) of wild type and *fveyuc10* (L3) obtained by qRT-PCR. (C) Expression level of *FveTARs* and *FveYUCs* in roots of wild type and *fveyuc10* (L3) obtained by qRT-PCR. *Gene11892* was used as the internal control. Data are means ±SD obtained from three biological replicates. (D) Morphology of wild type and the *fveyuc10* (L3) mutant at the same age. (E) Morphology and length of leaves of wild type and *fveyuc10* mutants (L2 and L3). Scale bar: 1 cm. No significant difference was found by Student’s *t*-test (*n*=21, 39, and 45 respectively). (F) Free auxin content in fruit of wild type and *fveyuc10* (L2 and L3 pooled together) at stage 4. **P<0.01, Student’s *t*-test, *n*=4.

To investigate the function of *FveYUC10* in strawberry, CRISPR/Cas9-mediated genome editing was applied to creating *fveyuc10* mutants in *F. vesca*. Briefly, two sgRNAs respectively driven by Arabidopsis *U6-26* and *U6-29* promoters were designed for *FveYUC10* (Fig. 5A). Three independent transgenic lines (L1–3) were obtained and analysed. CRISPR/Cas9-induced mutations were detected by Sanger sequencing of multiple clones of PCR-amplified fragments. The L1 and L2 lines each harbored homozygous mutations, while L3 contained bi-allelic mutations caused by both sgRNAs (Fig. 5A). In L1, the seventh amino acid was lost due to a 3 bp deletion at the sgRNA1 target site, and one nucleotide was inserted at the sgRNA2 site resulting in a truncated protein. In L2, the 2 bp insertion at the sgRNA1 site caused a premature stop codon. L3 had two alleles at the sgRNA1 and sgRNA2 sites. At the sgRNA1 site, one allele had a 13 bp insertion causing a premature stop codon; the other allele was the same as the L2 allele. At the sgRNA2 site, one of the two alleles had a seven-nucleotide deletion and the other a one-nucleotide deletion. Since all the mutations resulted in truncation of FveYUC10, the transcript level was determined by qRT-PCR in *fveyuc10* mutants. The transcript level was greatly reduced in fruit of *fveyuc10* (Fig. 5B), indicating potential nonsense-mediated decay. To examine whether other *FveYUCs* would be induced to compensate for the loss of *FveYUC10* in *fveyuc10* mutants, the expression levels of other *FveYUC*s were analysed by qRT-PCR in fruit, but their expression remained the same (Fig. 5B). Similar results were also obtained in roots (Fig. 5C). To summarize, the transgene of *CRISPR-Cas9* induced a 100% mutation frequency at the *FveYUC10* locus, and all the mutations should result in a complete loss of gene functions.

Next, the three *fveyuc10* mutants were grown together with wild type to observe the phenotypes. There was no significant difference in the length of leaf petioles and rachises (Fig. 5D, E), and the time from fruit set to ripening and the fruit size also remained the same. Furthermore, we measured free auxin contents in the entire fruit at stage 4. The average free auxin content in wild type fruit was 7.65 ng g^−1^ fresh weight. By contrast, the *fveyuc10* fruit contained free auxin of 3.04 ng g^−1^ fresh weight, accounting for roughly 40% of the wild type control (Fig. 5F, *P*<0.01). Taken together, loss of *FveYUC10* alone was able to reduce free auxin content, but not sufficiently to cause significant morphological defects.

## Discussion

Auxin plays critical roles in fruit initiation and fruit development in strawberry (Nitsch, 1950). Previously, we used RNA-seq to examine expression levels of auxin synthesis, transport, and signaling genes in wild strawberry (*F. vesca*) flower and fruit. An interesting finding was that fertilization-induced auxin biosynthesis genes were mainly expressed in the ghost (seed with embryo removed, consisting of seed coat and endosperm) rather than in the embryo within the seed (Kang *et al.*, 2013). However, we do not know if the auxin biosynthesis genes are expressed in the endosperm or seed coat. Higher tissue resolution of gene expression can be achieved by laser capture microdissection, single-cell sequencing, spatially resolved transcriptome profiling, or reporter gene expression (Brady *et al.*, 2007; Giacomello *et al.*, 2017). In the current study, we generated promoter::reporters in transgenic *F. vesca* to directly visualize auxin distribution and specific cell types of auxin biosynthesis gene expression. Specifically, *DR5ver2* reporter lines revealed that auxin accumulated in the chalazal seed coat and embryo within a seed as well as vasculatures in fruit receptacle (Fig. 1). In contrast, with the exception of *FveYUC4*, which is embryo-expressed and possibly functions in embryogenesis, *FveTAR1* and *FveYUC5*, *10*, *and 11* reporters showed endosperm expression (Fig. 3). The above observed endosperm expression of auxin biosynthesis genes combined with *DR2ver2*::*GUS* reporter expression in the chalazal seed coat suggests that the endosperm-synthesized auxin likely pools at the chalazal seed coat due to specific transport activities. Indeed, auxin efflux transporter genes *FvePIN1* and *FvePIN5* were previously shown, via *proFvePIN1::GUS* and *proFvePIN5::GUS* reporter genes, to be highly expressed in the chalazal end of the seed coat (Kang *et al.*, 2013), supporting this hypothesis.

### *DR5ver2::GUS* revealed auxin distribution and dynamics in strawberry fruit

Thus far, three versions of *DR5*, the synthetic auxin-responsive reporter, have been published, namely *DR5* (Ulmasov *et al.*, 1997), *DR5rev* (Friml *et al.*, 2002), and *DR5ver2* (Liao *et al.*, 2015). All three reporters report auxin signaling activity and hence are indirect indicators of auxin levels. Compared with *DR5* and *DR5rev*, *DR5ver2* displayed an expanded expression region with enhanced expression levels (Liao *et al.*, 2015) and was chosen for this study.

We showed that *DR5ver2::GUS* was specifically expressed in root tips and responded to auxin application in the root and fruit of *F. vesca* (Fig. 1), indicating that it is a good indicator of auxin activity in strawberry. The strong expression of *DR5ver2::GUS* in the receptacle vasculature is consistent with previous results using DR5 in the cultivated strawberry (Estrada-Johnson *et al.*, 2017) and indicates a possible traffic route of auxin from the endosperm, to the chalazal seed coat, and then to the receptacle via the vasculature connecting them. Expression of *DR5ver2::GUS* in the receptacle displayed an initial increase and then a reduction, a trend similar to the free auxin level change in the achenes at early fruit developmental stages (Symons *et al.*, 2012). This suggests that the receptacle at the receiving end of the auxin transport experiences a similar dynamic change in auxin levels, albeit at a much lower level based on prior measurement of free auxin (Nitsch, 1950, 1955; Dreher and Poovaiah, 1982; Symons *et al.*, 2012; Estrada-Johnson *et al.*, 2017). Nevertheless, the increase in auxin in both achenes and receptacle coincides with and may be responsible for achene and receptacle enlargement during early-stage fruit development.

### Auxin is mainly produced in the endosperms with potential roles for seed coat and fruit development

The formation of auxin gradients in embryo has drawn much attention as it is a good model system to study organogenesis (Benková *et al*., 2003), but where auxin is synthesized in other seed tissues other than embryo is not known. We showed that certain auxin biosynthesis genes were highly expressed in the endosperm (Fig. 3). For example, *FveYUC5* was specifically expressed in the chalazal endosperm. Moreover, three of the five genes studied, namely *FveTAR1*, *FveYUC10*, and *FveYUC11*, are more highly expressed in the endosperm, highlighting endosperm as the primary auxin biosynthesis sites within the seeds. In fact, some *YUCs* in other plant species have been discovered to be expressed in the endosperm, such as *AtYUC10* in Arabidopsis and *ZmYUC1* in maize (Bernardi *et al.*, 2012; Figueiredo *et al.*, 2015). Therefore, auxin production in the endosperm seems to be a universal phenomenon in plants. The biological function of the endosperm-derived auxin remains to be determined. One hypothesis is that endosperm-derived auxin is primarily targeted for endosperm, seed coat, and fruit development with no or little role in embryogenesis. In support of this hypothesis, auxin was found essential for endosperm development in Arabidopsis and maize (Bernardi *et al.*, 2012; Figueiredo *et al.*, 2015). In addition, endosperm-derived auxin was also important for seed coat growth (Figueiredo *et al.*, 2016). It is possible that endosperm-derived auxin is first transported to the seed coat (as shown by *DR5ver2* in Fig. 1) and then to the fruit to stimulate their respective growth.

### Auxin production and function in roots

*TARs* and *YUCs* play important roles in root development. Loss of their functions can cause moderate to very severe root defects in Arabidopsis (Cheng *et al.*, 2007; Stepanova *et al.*, 2008). This study provided a first look at the expression patterns of several *FveTARs* and *FveYUCs* in strawberry roots (Fig. 4). Auxin was considered to be produced in roots at significant levels with multiple sources in Arabidopsis (Ljung *et al.*, 2005). Consistent with this report, *FveTARs* and *FveYUCs* exhibited diverse and distinct expression patterns in roots, including vascular tissues, root tips, lateral root primordia, and cells at the base surrounding lateral root primordia (Fig. 4). According to RNA-seq and qRT-PCR, *FveYUC10*, *FveYUC3*, and *FveYUC6* were more abundantly expressed in roots, and may play important roles in root development. However, *FveYUC11*, which is expressed at an extremely low level in root based on RNA-seq (Fig. 2C) and qRT-PCR (Fig. 4F), showed a highly specific and unique tissue expression pattern at the base of the lateral primordium, indicating that *FveYUC11* likely facilitates auxin biosynthesis for lateral root primordial emergence and growth. Our data demonstrate that GUS reporters can reveal extremely useful gene expression information not provided by RNA-seq or qRT-PCR.

### Loss-of-function mutations in *FveYUC10* did not cause morphological phenotypes

We found that *FveYUC10* is the most abundantly expressed *YUC* gene in both seeds and roots in *F. vesca* (Kang *et al.*, 2013), which was also verified in an independent study (Liu *et al.*, 2014). In this study, *fveyuc10* mutants, created by the CRISPR/Cas9-mediated genome editing (Yang *et al.*, 2017), had a reduced level of auxin content in fruit (Fig. 5); however, no obvious morphological phenotypes were observed. In Arabidopsis, *YUC* single mutants usually do not exhibit obvious developmental defects due to functional redundancy (Cheng *et al.*, 2006; Chen *et al.*, 2014). However, other plant species may not have as many copies of *YUC*/*TAA* genes as Arabidopsis and single mutants could lead to significant defects, such as *FveYUC6* RNAi in wild strawberry, *de18* (defective endosperm18, *ZmYUC1*) and *spi1* (sparse inflorescence1) in maize, and *floozy* in petunia (Tobeña-Santamaria *et al*., 2002; Gallavotti *et al.*, 2008; Bernardi *et al.*, 2012; Liu *et al.*, 2014). The lack of phenotype in *fveyuc10* in *F. vesca* could be explained by functional redundancy among multiple *YUC* genes in the *F. vesca* genome, or the existence of a feedback loop, or alternatively biosynthesis pathways (Tivendale *et al.*, 2014) that could compensate for the loss of *FveYUC10*.

## Supplementary data

Supplementary data are available at *JXB* online.

Fig. S1. Validation of the positive transgenic plants examined by RT-PCR.

Fig. S2. Promoter sequences of *FveTARs* and *FveYUCs* used for plasmid construction.

Fig. S3. GUS staining of receptacles.

Fig. S4. Protein sequence of *FveYUC10*.

Fig. S5. Phenotypes of the 35S::*FveYUC10* transgenic plants in Arabidopsis.

Table S1. The list of primers used in this study.

Table S2. Expression patterns of *FveTARs* and *FveYUCs* in the transcriptome database represented by RPKM.

## Author contributions

CK, JF, and ZL conceived and designed the experiments. JF, CD, HL, and YH performed the experiments. CK, ZL, and JF wrote the paper. All authors have read and approved the manuscript.

## Supplementary Material

Supplementary Material
