# Supplementary material for: Reporter gene expression reveals precise auxin synthesis sites during fruit and root development in wild strawberry
Source: J Exp Bot. 2018 Oct 27;70(2):563–74. doi: 10.1093/jxb/ery384 (PMC6322568; doi:10.1093/jxb/ery384)
Supplement: Supplementary Material [file ery384_suppl_supplementary_material.pdf]

Reporter gene expression reveals precise auxin synthesis sites during fruit and root development in the wild strawberry

Running title: *TARs* and *YUCs* expression in strawberry

Jia Feng[1], Cheng Dai[2], Huifeng Luo[1], Yafan Han[1], Zhongchi Liu[1, 3*], Chunying Kang[1*]

1. Key Laboratory of Horticultural Plant Biology (Ministry of Education), College of Horticulture and Forestry Sciences, Huazhong Agricultural University, Wuhan, 430070, China
2. National Key Laboratory of Crop Genetic Improvement, College of Plant Science and Technology, Huazhong Agricultural University, Wuhan, 430070, China
3. Department of Cell Biology and Molecular Genetics, University of Maryland, College Park, MD, 20742, USA

*To whom correspondence should be addressed. Tel. +86 13871595425. E-mail: ckang@mail.hzau.edu.cn, zliu@umd.edu.

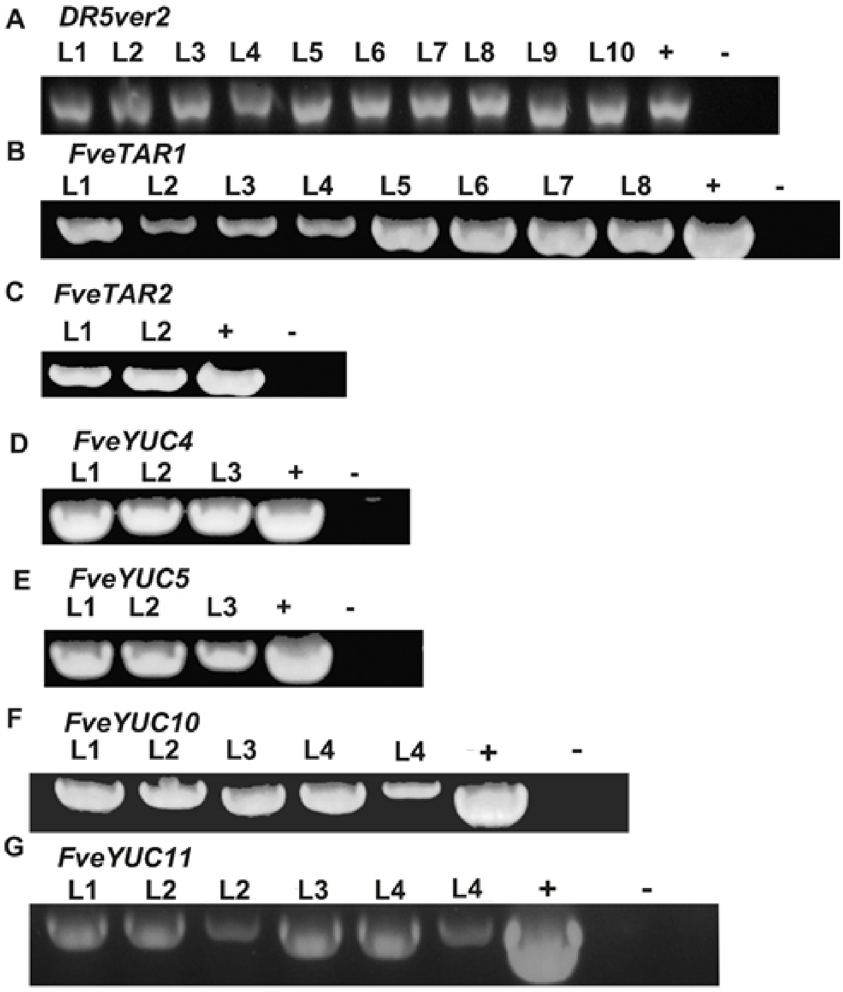

Figure S1. Validation of the positive transgenic plants examined by RT-PCR.

(A) Amplification of the *DR5ver2* fragment (464 bp) in *DR5ver2::*GUS reporter lines by RT-PCR. (B) and (C) Amplification of the *FveTARs-GFP* fragments (719 and 801 bp) in *FveTARs::GFP-GUS* reporter lines by RT-PCR, respectively. (D) to (G) Amplification of the *FveYUCs-GFP* fragments (770, 587, 905, and 708 bp) in *FveYUCs::GFP-GUS* reporter lines by RT-PCR, respectively. The plasmid was used as the positive control, and the wild type Hawaii 4 was used as the negative control. In (F) and (G), the results of two biological replicates were shown for three lines (*FveYUC10::GFP-GUS*-L4, *FveYUC11::GFP-GUS*-L2 and L4). The primers are listed in Table S1.

Figure S2. Promoter sequences of *FveTARs* and *FveYUCs* used for plasmid construction.

>*FveTAR1*-promoter sequence

CGACATTGAAGTTTGTCGCAGATTCCTTTCCAGAAATTCATGCATCCGTTTTCAAAATGTGTGGCTAGC
AAATATCGATAGTGAAATAGGTCGAATTACATATATACCTTTTCAAAAATTCACGTATCCGTTTTCAAAAT
TAGTGGATAGCAAACATTGCATATACCTTTTTAGAAATTCATGTATCCGTTTCAACATAAGAGGCCAACA
AACATTGACATTGAAGTTGGTCACAGATACCTCTCCAAAAATTCATGCATCCGTTTTCAAAATAAGTCG
CCAGCAAACATCGACAGTGAAGTTGGTCGCATATATCTTTCCAGAAAGTCATGTATCCGTTTTAAAATT
AAATGGCTAGCAAATATCGACATTAAAGTTGGTCGAATTGTAGATAAGGATGGTTAAGCTAGGGGATAA
GCGTTTCACTAATTTCAAGCCACATTCCCAAATATTTATGCATCCGTTTTCAAAATAAAAGGCTAGCAA
ACATCGACATTCAAGTTGGTCGAACCGCATATAAAATTGGTTAAACTAAGGAACAAGCGTTTCACTAG
TTTCAGGCGACCTTCCCAAAAAATCAAGCATCTGTTTTCAAAACAAATGGCTAGCAAACATCAACATT
GAGGTCGGTTGAATTGCAAATGAGATTTTCACAATTCGCATAAGAGCGTTAGAGAAAATTCATGCGTAA
TTTATGGAAATCAATAGGTAACGCACTAGAGTATGTTGACTAGGGACAATATTTCATAATGTTTCACACA
TACTAACGCAACAACTTCAACAAAATCACGTGAGTGTCATAATATAAATTTCAGTATTTCTATGACGAC
CGTCTATAAAATCGAACACGAAGTAATCAGAGACAACCAAAATTCCAATCACATTTTTGCTCTCTTATC
AAGCCGAAAGTTTTTACCTCCAACTAAGGCAGGTAGACATTGAAGATCAATTGTTGAGCAAATCTATC
CAAGCATATACACCACTACACCAGCCATAGAGATTCATCCATTTCCCTGCCAATTCAATCAAACTAGAG
TTCCTATGGCTAGGATCTACATCAACTTTCTCTTTTCCTCTTAACCAAACACCAACTAATTGTGATTCTT
TTAGTTGGAAACAAATACAATGTGTCCTCTTAACCAAACTCTTTCTTCTGGAATCTGGATTGATGAAAT
AAAACCCAGTGTCCTTCATGAACCACCTTCTTCGAGTTTTTGATTCGTTTCAACTCATTGCTCTTCTGCT
TCACTCTTTTTCTCTGATGAATCGAGGCACTGCAATTTCAGAGGCCTTTTAGATTCTCCATGCTGCTTTC
TCTTTCTGTTCTTCTTTACTGTTAGCTTTGTGTAGCTCATTCAACACTCCAAAACATTCCAAGTTTTCTT
CAACGTTACAAGCTTAGAAAAACAACATTATGAAAATATTGGGCTTCATGGCCCACTAAGAGTCCACA
ATGTTGCATCCCATGGCCCAATTACTTTATGTCCAGGAAAGAGCTCCAATTCATCAACTGCCGCCAACT
CATCGCCAAGTTCATGGTCTTCTGCAATTCATCAGTTACTTCCTTTTCTGTTTTGCATTATCTGTATTTTC
TTTTTCTGGGAATAAATTCAATGTCTTGGGAAAACTCACATGGAACAGCAGCATTCTCTATAAACCCAG
AATTTTGGGGTGTCCTTCCAAACTACCAATTAAACGTCTTTGACATGAATCCAAGATGCAGTTTCAAAT
TTTGTCATGGTGGAATTGACTGGAGTAGCATGTACAATGTGACTCCAGAAGACCAGTTTATTCAACTTG
AATCCAGAAGACCCGCTTCTGCAAGCGTTACAGTTCGTTCTTCAACATCCATTTTCTCCTGGGTTTCGC
TATAAAAGGATTAAAAGCCACCTCAAGTTTTTCCAGTTCCTCACCTTGTTCCAAAGCATCCTCTGTTTT
CATTTCCTCTGTTTCAGCATTCACTTCTTTCAAGTTTCAACTACTTCCCTTTCCATAGCTCTACAAA

>*FveTAR2*-promoter sequence

ACCGCAAACCAATCTTATCCAGAAACAACACATGCCCTAAGAGATCGCCGACCTTCCCTGCAGCCTCT
TCTGTGAACAAACGTGGTGGGAGTCCAAGGATCTCAACCCACACCGACATAGAGGAGATCAGAACAG
TGGTCACCTTAGAGAGACCATCACACTCTGAAAGCATGAAGTTGGATCGTCCAAAGAACCATGGACCT
CCTTGGATCGGATAGCGATGATCTTCAGGGCGAGAGAACTTGAGCAAATATCGATCTCCCACCGAACG
TACCTGGAGACGGTTATGGAGATCCCAGACGGAAGTCAGGGTGTCGATCACCACCTTCGGGTCTGGG
AGACGCTTCTTCATAAACAACAGCCTACCAATGGCAAGAGAGGGAGCCAAGGCAGTCGGAGGTCGTG
GAGTCACTCAGGGAGTGGCCGGACTGCCCTCACGAATGGTGAGAGCAGTGGCGAAATGGATTGAAAC
CTCATCAGTCATGGTGAGCAAACAGGGAATGGGTGCTGAGCGGTAGAGAAAAATGAAACCCTAGGGA
AGCAAAAACCCTAGCAGCGCTCTCTATTAACATGCAATAGAGTTAAAAATGGCCCCTCATACGTGATAA
CCTTTTCTTTTAAAAAAAAAGGACGGAAAATTTTATTCAGACTGGATAAACATACGTGATAACTTAATA
ACTCATTATTGAACTTTAAATCGTTATAAAATAAAATAACAGAAGTTACAGATTTTCTTACGACGAAGAT
TATCATCCAATACAATTGATAGTTCGCTGAAGTAACTCCCAAACATCAGTTAAAGGTTACATATGTAGTG
TCCAATCACTTAACTTGAGTTACGGTTCTTTAGGTTATGAACCTAAATTAAGAAAATTGCATTCTGGATT
AGACACCAAGCCAAAAAAAAAAACAAAGCACAGAATTACCAAGGCACACAAACGTTCCACTATGAAA
CGTAATTAGCAGGAACCTCATCTGTCACCATCTCCCTCTCCTAGTTCCAGTCCACATCAAACTCCAATT
CGTTACAACTCAAAAATATCAGACTTATTAACCAAAATGGATTGAAACCAAATACTCAAGAGACCAAA
CCAAAATAGAAAAAATATAAAACAGTACAAAGGAGAGAAGAAACGAATAGAAGAAAAAAAAAAATGA
TGGGGGGATCAGAGAACGGAGGGATCAGAAAAAGCAAACAGAAAAATTGCAGACAGAGCATGCAGT
AAGAGGCAGGCTTTATTTCCACGCCCTTAATTAACCCATATACAAAACCTGCCGTGTATTTATATTCACC
TTTGGAACAATGAATCTATTACTACCTCTACAGTTCTACTCTTAATTGGGTGTTCTTGTTTTCGAGGGGC
GAGATCTAGCTTCGGTAACCGTTCTGGGATTTCTTGGTCCCTGAAAAAAATCTCCGGCCCGGAAGTGT
TTTCAACTCTCCGGTGGCATTTCATCACCTACCCACCTCATCAAGTTTCTTCCTTTTCTGTTTTCCATTAT
CTGATTTTCACAAATTCTTTACTGTCTTTGAAAAAAATCACATGCAAGAGTAATCTAGAGTGTCTTTCCT
AACTGTCAGTGAATTCTCTTCGACATATACCCAACTTCCAAACAATCTATTTCAAGATTGATCATGGTTG
GATTAACTGACCTAGTTTGTTCAATTTCAATCTGTGTAAGCCTTAAAGTTCACTCTTTACCACCCATTTG
TCACTTGAAACACTTTTTTGTTTTCATCTCCTGGGTTTCTCTATAAAACCCGCCTCAAGTTTCTCTATAA
AACCCGCCTCAAGTTTCTCTCCACTTCCTCACATTCACTCACTCTGTTTTCATTGCTCTGTTTTCAGTAC
TCTGTTGCTCTGTTTTCAGTACTCAAAATATGACCAAATATGAGAGCATGTTCACA

>*FveYUC4*-promoter sequence

GTATCGTCGAGTTTATCTTCAACTGCAACATAATAACTAGGAAAGAAGCATTATTAGATATTTAGATATGT
AAACATGTTATATATCTAATGATGTTTCCATTATATTACTGATGTGACAAAATAAATAGTCACAATTAGAG

GAGTAATCTTTGCTAGAAGATGTCTATAAATATACGTACATTATAGAGTTTTTGGTATTATTGAGGGAATA
CTTCTAGATGCTTATTGTAATTAGGATTTATGTTTTATGTCTTTTTTTTATATTCGACAGTTTTATTAACGA
AAATATGAGACAGCCACACCAACCTTTTATTAAAAAAAAGAAAAAAATATCAATTCTGCCAGGCGGGA
GACGGCAAGTATAATGACATGTGGCGCTTGAGAAAAAGTAGTATTCGATCAACGGCTGTTGACGCAAG
TAATACCTCTTTTCTCTGCTTCCTACAATGCAACAAATTAACAACTACTCCCTTTGCTGACTTGGCAGAC
CACGTGGCAAAATATCACGTCTCCAAGGAGCTAATCAACCCCATGTCCTTCACTCCACGCGCCTTGTC
AACATCCATTGAACACTCTCATCTTCCTTTCTTGTTTGCTAAAAAGAAAGAAAAATATCTTTTTCTTGTT
CCTTTTGTGTAGAATGTTTCCTTTTTTTTTTTTTTTCTCAAATAATCTGATTAAAGTGAACAAGTGTTTAT
ATCTGAAAAATGAACAGATATGATTAAGTAGAACAAGTGTTTATATCTGAAAAATGAAGAAAGTATTAT
GTGAATTAAAATAGTACTAAAATGTGCAATAATTTTTTGACTTAAAACTAATATGATTCTTTTTTATGTTA
ATTCAAAATAGTAGGCTTCAGTTAGACACATGCAACTTGTAGAAAGAAAAAATGATTAAGTAGCTAAA
TGTTTTTTATTTATTCAAAATCACGGAATTTCAAAGGTCTAGACCCAATAATTAATCTGTTGGGGGCCCG
GACAATCAGCAGGACGTGCAGCCCCTCTCCTGATCATTTTTGACAGTGTCATGGTTCGAATATAAGTTT
GGGAGGATACTCAATTAGACAATAATCATTAAACTACATTGCAGTGTTAGATAATCAATTAAAAGAGAA
AATAGTGGGTCAAAATTCGAGAGCAACTGTTCAAAAATAAATGAATTCTATCGATGAAATTTAGTTATA
GACCACGTTCAGTGTATTTGTAGTTAAAACTAATTGTAATTCTGAAATACTGAAACTGTATAAGTATATA
GTGTATAATAGAAGTCTTTTCATCCGCCAGTACTTCACTTACTGACCAATATTCACTTTGTGTCCAATTC
AAACACACGGGGACTATCTTTACTAGAACAAAGCTTCTCGACCTCGTCTTGGATTAGTAATCATCTATG
ATTTTGAACAATTCAAACAAAACCTGCGTAGAGCGTGTAAAACCACGGCTTCAAACCGCGAGTGTGG
CATCCCCGTTCTTCTCAAATCTAACTCGTATCAAAGAGCACCATAGAAATATGTATTTATATCTTCCCCTT
CCTCCATTGCATAAAGCCTACTACTATTACCTCTCTGCACACAGAGCAGAAACTAGCCTTGAGAAACA
GAGCCAAATCCTTTTCCTTAACCTTCACGCCTCCATTAAAAGCAGCTTAAACCTTTCTCTCTATCTCTGC
TCCTCTCTCATAATTACTCTGTTTCTCTCGCTCTGTCTTCGACACATTTAAAAAGGGTCTCTCTCGGTCC
TCCACAGAGCCCCTCATCACTGATTCGAAGCTTGCCTCCTTTCTTTAAACCCCTCTTTGAATTCTTTGAA
TTCCAAGTACCTTGTTTTTTAGTTTCCCACCTCATCTTTCCTCAGCAAAACAGAGCAAGAGAAAACAG
AGCCTTTAAAACAGAGCAACTTACTCTCTGTTTTCCCTATCCCAAATCTTCTTTCTTTCTTTCTTTCACT
TCCCCTCATT

>*FveYUC5*-promoter sequence

GCTTCTTCCTCAAGTGGCTCCTAGGAGGCGGAAGACCTCTGTTAGCGAGGAAGCATTTCATGGTTTTG
GACCATTTGGTTGTTGGGTGATGTGTTGTGGTTGATTTGGTTGGGAAAGAAGATCTTTTCCTCCGACGA
AGATTGAAAACAACAAATGTGGCTTCTCCCAACGAGATGGTGCTCGTGTTGGTGGCAATCCATGCACC
AATCCTGGCGTACTTCAACAGTGGCAGTGTGGACGGTGTCGGCGTCATGGTTGGTTTTCTTCATGTGTG
TCCTGCTTGGCGTCGACCTAGTGGGAACTGGGCTAATGTTTGTTACTTGGGCTTGAGTTCTACTAGGTT
GACCAGAGGTAAAACATTTGGGCTTCCTAAATTTTGGTTTCAGATTTGGGATCTAGGTGGCTTTACGAT
AAAAATTTGGGTCCTAAGAGTTTACTTAGTTCGGTACCAGTTTTTTTCTTGGGATCAATACTTGCGAGG
AACTGCCGGGATTATTGATCGAAGGTTGTCAGTGAGTCTAATGGATGCCGCGGAAAAAATTTATTATCAG
TATTTTATTAGTTCACTCAATAGGTAATTAAGTTGTATTACATTTTAGCGATGTCTCTTTTAGACGACTCA
CTCTCTTCATGGAGGCAATTCAATTTGTAGCTGTGGGCTTTTGGCAGAGATTGTTTATAGATCTACGGTC
GGTGGGTGGGGGGTCTCGCATAGGCTTCTTCCTCAATTGGCTCCTAGGAGGCGGAAGACCTCTGTTAG
CGAGGAAGCATTTCGTGGTTTTGGACCATTTGGTTGTTGGGTGATGTGTTGTGGTTGATTTGGTTGGGA
AAGAAGATCTTTTTCTCTGACAAAGATTGAAAACAACAAATGTGGCTTCTCCCAACGAGATGGTGCTC
GTGTTGGTGGCAATCCACCAATCCCAGCGTACTTCAACAGTGATGGCGTGGTTGGTGTCGGCACCATG
GTTGATTGTCTTCATGTGTGTCCTGCTTGGGGTCGACCTACTGGCAACTGGGCTCATGTTTGTTACTTG
GGCTTCAGTTCTAGTAGGTTGACTAGAGGCAAAACATTTGGGCTTCCTAAATTTTGGTTTCGGATTTGG
GATCTAGGTGGCTTTATGATAAAAAATTTGGGTCCCAAGAGGTTTCTTAGTTCGGTATCAATTTTTTTCGT
GGGATCAATACTTGCGAGGAACTGCCGGGATTATTGATCGAAGGTTGTCAACGAGTATTCTAATGGATG
CCGCAGAAAAATTTATTGTCAGTATTTTATTAGTTCATTCAATAGGCTAGGTAACAATTAAGTTGTATTAC
GTTTTAGCGGTATCTCTTTTAGATTGCTCTGCAAGGGTCACTGTGTAATATGGCTTGTGCTTATTAAATA
AAATCGTCTAACCTCTTTTCAATTTAAAAAAAAAAAAAAAAAAAAAAGACCCTGCCATGTTGATGTTAA
CATTCAAAATTTTAGATAATCAGTTTCCATTTTCTATTATTGCATTTAAAATAGCCGCGGAATCTTTTAGA
TTCATTGTGTGTGATATATAGTGTAGCTTTGTTAGAAGATTTGGCATTTATGTAATCAGATACGACGTATC
CATCTTTATAGAATCATTCTACACAAATGCAAGTTTATTTGGTAAAAAACTGAAACAAAATCTACCTTTT
TACACAACTGTTCCTATCCATTAACAGTGCAACTAACTATGAAATCTTACGGCTTAGATTGTCAGCTAAT
TATCTCTACTCACATTTTAGTCCATACATTTTAGACAATGTTAAATGAACTTATATAGAATCAAATTCCCA
CTGCTAGATGTGGATACCACTAGCTTCCCAAATGTGCATCTATGTCTTCAACATGCATTAATTGTCCTAG
GATAGGTATTAGCAGTAGTATATAAACATGCAAGCACAATACATATCAGTTCATGATGATAGGTATCTGCT
AAATCTACACACTCTTGTCATCTAGAA

>*FveYUC10*-promoter sequence

CACACCGCATCTTCCTAGTTTTTGTTACGAACTTACGATTTCGATTGCAGGACAAATGACATGATGCGA
CGTTGTATTATCCGGTTTATGGTTTTGGATTGAAATTAATTGCAAGAACATGATCGTCGAGCCCTAACTA
TCCCTACATGATCGAGAATTTTCTTTTTTAGTAACCCTCCTAAGAACGAGGTTATTTAATGAAAATAATA
AAAAGAGAAGAAGGAGACAAACACCGAAACCTCTCATACAAATTAAGAAACAAACACTAACGAGAT
AGGAAACTAGGGACTTTGCATAGACATTCATATATAATTTCAAGAATTAAATTCAGTTTACCCCCTTTGG
TTTGGGGGTGACTTCATGTTAGTCCCTACATTTTTATTTTCATCAGTTTACCCCTTGAACTCTTCAATTTT
TGTCTGCCGTGCCCAAATTCTCATATTCTGTTTGAATTAACCTTTAATTATCAGCAATTAAGGTCCGATTT
GGACATATAAGGTCCGATTTACCCAAATTCTAGATATTGGCCTCACAGTTAATGTTAAAATTATTAACAG

TTAACGTCCGATTTAGACAGAATATAAGATATTTGGTCACGCTTGAGGAAAATTCAAAAGTTTGAGGGG
TAAACTGATGAAATTGAAAGTATAGGGATTAACATGAAGTAACTCTCAAACCTCATGGGGGTAAACTG
AATTTAATTCTAATTTCAAATATAAATTGCAAAATGAGGTAATTCATAGTTAATTGTTAACAATTCCTCAT
TAATTTTGACTTCAATCCAAATTTTATTTTCCTTCCTCCAAACGGAGAAATTTTTAAGTGCTACGGGAGG
ACCACATGACAGTCTGACGTGGTCCTACATTCCAATCAAATTTAGACATGTGGATTTTTTACAACTAAA
ATATTAACTAATATTTTTTATTTTTTGTGAAATGACATTCATGGGTATTTAGCAAGTTCAACCTAGGGTTT
AGGGTTTAGAGTTTAGGGTTAGGGTATAGGGTTTAGGGTTTAGGGTTTAGGGTTTAGGGTTTAGGGTTT
AGGGATTAGGGATTAGGGATTAGGGTTTAGTATTTAAAAAACAACATAAAACCCAAAATAGTCTTTAAC
AAAATAGTTTAAATAATTTTTCATGACATGTCTAAATTTAATTGGAATATAGAACCACGATTACCACGTT
GTTCTTCTGTAGCACTGAAAAATTTCTCCTCCAAACGGAGCACTAAGATTTTCAGATGGAGCATCTGCT
ATAGAAAGTAGAAATATCAAATATATATAAAAGCCAGCCAACCCACTCTCAATCTTTCTGCTCATGCTAA
CTAAGCTAGCTATATATAGAGAGTTATAGAGACTAAGATTAATCCCCACCTTGCTAAAAGTGCACACCA
CTCCTATACATCACCGCAAA

>FveYUC11-promoter sequence:

TAGGCTTAGAATTGGGCTTCGACTTCTTCTAGAAAGTTGTTCGAAATGTTGAGAAGAAGATTCTGTCA
AATTTTGGTGGTGATTGGAGGTGGCCGAAAGTTTCTGCAGTTTTTTTTTTCAAAAACCAGCAACTTTA
GCCGACGATATTTGAAGGTTTATTAAGGTTTAGCCGACGAATTATGGCATATTTCGTCGGCTATAGTT
ATTTTTTAAATTTTTTATAAGGTTTAGCCGACGAAACAGACTATATTTCGTCGGCTATAGTTATTTTTT
TAATTTTTTTATAAGGTTTAGCCGACGAAACATACTATATTTCGTCGGCTATAGTCTGAGAAAAAAAT
TTTTATTACAGACTTTAGCCGACGAATATCTTAATTGTTTCGTCGACTAAAGTCTGGGAAAAAAAACCG
ACGACATGTTTTTCATCGGCTAAACTGCGGGACTATAGCCGACGAAATTTTTTTTTTCGTCGGCTAAA
GTCATCACATACGACCTTTTCCCGACGAAATCTTAACCGACGATGGCTCGTCGGCTAAGATCTTAGAC
GACGAATTAAGCTAACAGGCCGACGAAAATTTTTCGTCGGCTAAAGTGCTTGTCCTGGTAGTGTTAC
CAGACTACAGAAAATTTTGAACTGCTGCAACAACCTCAGGTGGCTTGACTCCTAATCTACTTAAACA
ACAAATATTTCCCAAAATGGGAAGTCGAAAACTATAATCTCTTATGATTGTGATTTGTCGATACCTAA
ACTGACTTAACATCTGAGAAGACCGACAACCCTGGTCTATCCTCGAACGTAGTGTTTGTTTTGACAAT
TATAACGGTACAGGAAGAGCACAAAACTTGTGTTCCACTTAATCGCGTTATCATGAGCACACCTAAA
ACAATATTCCATTTATTGATGATGGTTGAGGTAGAGATCATAATCGAGCTGCACTTATTTCGGTTAAA
TTTTAGACACTAAACTGTCACTGTACGTACTACCACTTCTGTTTTTGACGTCACTTATTTCAGTTAAGA
TAACAACTTTAGACATCACCAACTATCAAAATGTGATTAGTGTTTCAAGATGAATTATTGAGAAAAT
GATAGATAGTAAAGAGAATGGATAACCATCATCTTATTTATTGATATGAACCATTTCTATAGAGATT
ACATAGCATCATAAGGTGTTGTATAAAGAAATATATCTTTGATATCATATCTCTATATAGAATCGTGA
TTCCTTGTTTGACTAGCATTAGATCAAAACACACACATATAGAATATTCGTGCAACAATTAGCAATAT
GTATCATATAGAATGAATATATTAGCATACGGCGATAATTCCAACTTTATGTCATTATCCTTTTTTGTT
TTGAAAACTATATGCTCTTTATCTTGTTGGATATATGTCATTGTAAATGGATAGAAAATGTATCTGGT
AAGTATGCAAAAGAGTAAATGGTTATTTCTGAAAGCCTTGCTTCCAATATTACTATAAAAGACTAAT
ATCAAACAAATTGTTAATTTGTTACCATAAAAGACGATTTCAGGAAAACAAGATTAATTTGTCACCG
TAAAAGTTGATTCCAAAAAAAGGCAAAACTCAACAAAATCCAACGAATGTTCAACAAAATATATTCA
ACATTAACAAAGCACAGTAACCACCTAGCTACTAAACATAAAACCTTAGCAATTACCACTAATACCA
ACTCAGCCAAAGTAACAATAAACCATAACTGTTAATGGACTCGCTGAAGTCATCCTCGAGCTTCTGA
AACACCACCTCACAATTTGCATGCATCAAAAGTGGATGGTGGTGATCGATGAGTGTAACGGTGATCT
TTGTGATTTTAAACAAATAAAATATTCTGCACTTCTAGCTTCAATGGCGACAGTCTGTCAAGTCCTCT
CTTCTTCCTCACTAGTATAAATGGTCTTCCTCTTTGCTGTGGTTCATCATCCCCAGCAAGCCATTGATC
TTAAGAGAAAGGGAGAAAGATTAGAAGTGAGGCAAA

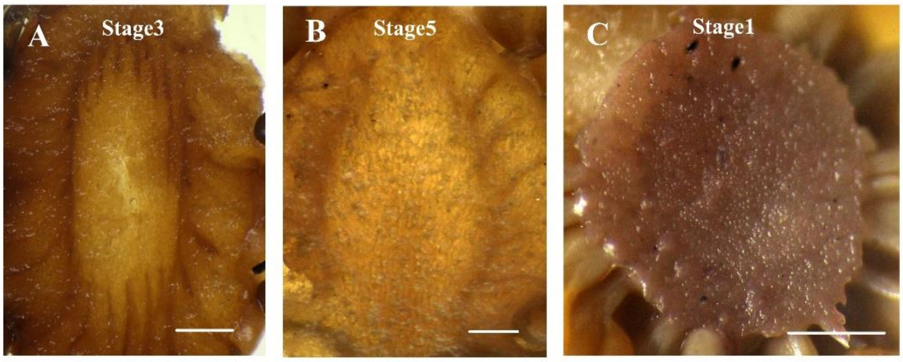

Figure S3. GUS staining of receptacles.

(A) *GUS* expression of *FveTAR1::GUS* in receptacle (longitudinal section) at stage 3. (B) *GUS* expression of *FveYUC5::GUS* in receptacle (longitudinal section) at stage 5. (C) *GUS* expression of *FveYUC10::GUS* in receptacle (cross section) at stage 1. Scale bars: 2 mm.

Figure S4. Protein sequence of FveYUC10

> FveYUC10

MPEAAVIIVGAGPSGLAVAGCLSRLEIPYLLLEREDCFASLWKKYSYDRLHLHLQKQFCELPHMSFPSSCPT
YVPKKQFIQYLDDYVAHFKISPMYQRNVESATYDEGSERWVVKAMNNDEGCGGEVEVFLGRFLVVATGE
ATNPYVPEIEGLSSFDGEVLHSTRFKSGVEFKNKNVLVVGSGNSGMEIALDLANHGARTSIIVRSPVHFLSK
RMVYLALVLLRYLSLSKVDTLMVLLSKLVYGDLAKYGIARPKEGPFFMKIKYGKYPAIDVGTCSKIKSGEI
QVLPTEIGSIRGNDVKLKNGKSYQFDSIVFCTGFKRSTHLWLKGDDYLLKEDGLPRPSFPNHWKGKNGLF
CVGLSRRGLYGSSEDAQNIANDIQSSL*

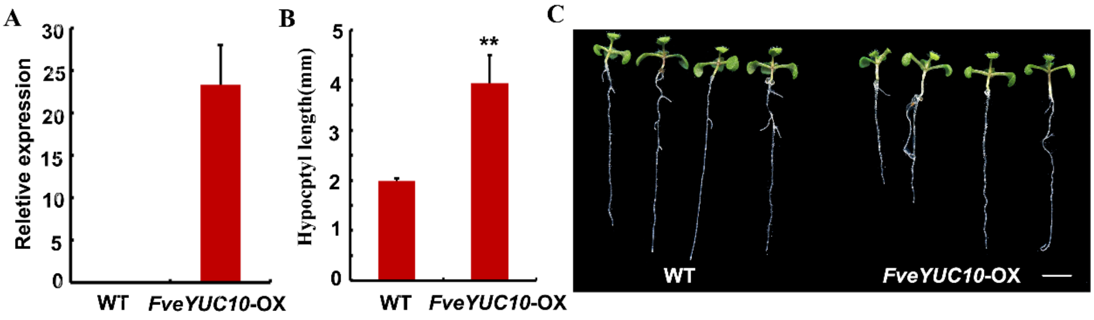

Figure S5. Phenotypes of the 35S::*FveYUC10* transgenic plants in *Arabidopsis*.

(A) Expression level of *FveYUC10* in the T2 *35S::FveYUC10* seedlings. *Actin* (At3g18780) was used as the internal control. Data are means±SD obtained from three biological replicates. (B) Measurement of the hypocotyl length of 35S::*FveYUC10* in the T2 generation and the wild type *Arabidopsis* (WT). n = 40. **, $P < 0.01$, student's *t*-test. (C) Images of the 10-day-old seedlings of 35S::*FveYUC10* in the T2 generation and WT grown on the MS medium. Scale bar: 5 mm.

Table S1. The list of primers used in this study.

Primers used for qRT-PCR		
Gene name	Direction	Primer sequence (5'-3')
FveTAR1	Forward	AAGTAGTGCCAACTCGCAACCT
	Reverse	AGGCATCAGGCAAGGACTGG
FveTAR2	Forward	CTTGTGCTGTCTCTGGCGCT
	Reverse	TCTGGGTTGCGTGAGCTTCC
FveYUC2	Forward	AAGAGCGGCGAGATGTTCCG
	Reverse	AGCAACCACATCGACAGCCC
FveYUC3	Forward	GACGCGTTGAACTTGTCGGC
	Reverse	CCTTGTGAACCCGACTGCGT
FveYUC4	Forward	GCTTCAACCAGGCCGTCCAA
	Reverse	CCGGTGGCCACAATAAGCCA
FveYUC5	Forward	GCACTGTGCTTAAGTGGCTCA
	Reverse	CACGCCTTGAGAACCCAGCA
FveYUC6	Forward	ACTGCAGGGTCCGAAAACGG
	Reverse	GCCCGAATTTCCACACCCCA
FveYUC7	Forward	GGCGGGTGAAGACCATTGCT
	Reverse	GCATGACATGGCCGCCAAAG
FveYUC10	Forward	TTCTGCACTGGGTTCAAGAG
	Reverse	GAGCATCCTCACTAGATCCA
FveYUC11	Forward	CCTCCGGCATTGCAACCTCA
	Reverse	ATGCGGCATCATCGGCAGAG
GUS	Forward	CTGGCAACCGGGTGAAGGTT
	Reverse	ACTGTTCGCCCTTCACTGCC
Gene11892	Forward	AGCCTAACGCAGAGGTTCCAAA
	Reverse	GCAGCCCACATTGAAGGGTCTATAGT
Actin (At3g18780)	Forward	TCCCTCAGCACATTCCAGCA
	Reverse	GATCCCATTCATAAAACCCCAGC

Primers used for making the constructs		
**Construct**	**Primer name**	**Primer sequence (5'-3')**
FveTAR1::GFP-GUS	Gene37056-pro-F-B1	GGGGACAAGTTTGTACAAAAAAGCAGGCTCGACATTGAAGTTTGTCGCAGAT
	Gene37056-pro-R-B2	GGGGACCACTTTGTACAAGAAAGCTGGGTTTTTGTAGAGCTATGGAAAGGGAA
FveTAR2::GFP-GUS	Gene31790-pro-F-B1	GGGGACAAGTTTGTACAAAAAAGCAGGCTACCGCAAACCAATCTTATCCAG
	Gene31790-pro-R-B2	GGGGACCACTTTGTACAAGAAAGCTGGGTTGTGAACATGCTCTCATATTTGGT
FveYUC4::GFP-GUS	Gene11728-pro-F-B1	GGGGACAAGTTTGTACAAAAAAGCAGGCTGTATCGTCGAGTTTATCTTC
	Gene11728-pro-R-B2	GGGGACCACTTTGTACAAGAAAGCTGGGTAATGAGGGGAAGTGAAAGAAAG
FveYUC5pro::GUS	32686-F	GGGGACAAGTTTGTACAAAAAAGCAGGCTGCTTCTTCCTCAAGTGGCTCC
	32686-R	GGGGACCACTTTGTACAAGAAAGCTGGGTTTCTAGATGACAAGAGTGTGTA
FveYUC10::GFP-GUS	Gene27796p-attB1	GGGGACAAGTTTGTACAAAAAAGCAGGCTGCCACACCGCATCTTCCTAGTTT
	27796-R	GGGGACCACTTTGTACAAGAAAGCTGGGTTTTGCGGTGATGTATAGGAGTG
FveYUC11::GFP-GUS	Gene06886-pro-F-B1	GGGGACAAGTTTGTACAAAAAAGCAGGCTTAGGCTTAGAATTGGGCTTCG
	Gene06886-pro-R-B2	GGGGACCACTTTGTACAAGAAAGCTGGGTTTTGCCTCACTTCTAATCTTTC
DR5ver2::GUS	DR5ver2-attb1-F	GGGGACAAGTTTGTACAAAAAAGCAGGCTGCGGATCCAAGCTTCCGACACCGA
	DR5ver2-attb2-R	GGGGACCACTTTGTACAAGAAAGCTGGGTCCTGCAGTGTAATTGTAACTGTA
FveYUC10-ox	YUC10-OX-F	TCTTCACTGTTGATACATATGATGCCGGAAGCGGCGGTGATA
	YUC10-OX-R	GCCCTTGCTCACCATGAATTCCAGAGATGACTGGATGTCGT
FveYUC10-CRISPR	FvYUC10-DT1-BsF	ATATATGGTCTCGATTGAAGCGGCGGTGATAATAGTGTT
	FvYUC10-DT1-F0	TGAAGCGGCGGTGATAATAGTGTTTTAGAGCTAGAAATAGC
	FvYUC10-DT2-R0	AACTACTTGGCCAGGTCTCCATCAATCTCTTAGTCGACTCTAC
	FvYUC10-DT2-BsR	ATTATTGGTCTCGAAACTACTTGGCCAGGTCTCCATCAA

**Primers used for validating the transgenic lines in strawberry**		
Gene name	**Primer name**	**Primer sequence (5'-3')**
FveTAR1	Forward	CTCACATGGAACAGCAGCATT
	Reverse	AAGAAGATGGTGCGCTCCTG
FveTAR2	Forward	CATTTCATCACCTACCCACCT
	Reverse	AAGAAGATGGTGCGCTCCTG
FveYUC4	Forward	CTTCCTCCATTGCATAAAGCC
	Reverse	AAGAAGATGGTGCGCTCCTG
FveYUC5	Forward	CTTACGGCTTAGATTGTCAGC
	Reverse	AAGAAGATGGTGCGCTCCTG
FveYUC10	Forward	TTTAAGTGCTACGGGAGGACC
	Reverse	AAGAAGATGGTGCGCTCCTG
FveYUC11	Forward	CAAAGCACAGTAACCACCTAG
	Reverse	AAGAAGATGGTGCGCTCCTG

Table S2. Expression patterns of *FveTARs* and *FveYUCs* in the transcriptome database represented by RPKM.

RNA-seq data are from a few published studies as summarized in Li *et al.*, 2018. Expression level of each gene was color-coded to show the relative expression level among different tissues. Red indicates that this gene is more highly expressed in this particular tissue compared to other tissues.

	TAA1	*TAR1*	*TAR2*	*TAR3*	*TAR4*	*YUC1*	*YUC2*	*YUC3*	*YUC4*	*YUC5*	*YUC6*	*YUC7*	*YUC10*	*YUC11*
**SAM**	0.0	0.0	4.4	0.0	22.2	0.0	3.3	0.0	6.3	0.0	0.1	0.0	3.5	0.0
**SFM**	0.0	0.0	22.8	0.0	23.8	0.0	3.5	0.0	9.8	0.0	0.1	0.0	7.3	0.0
**REM**	0.0	0.0	7.5	0.0	15.2	0.0	2.0	0.0	6.8	0.0	0.1	0.0	3.1	0.0
**Ovule1**	0.0	0.1	0.9	0.0	64.0	0.0	0.9	0.1	0.2	0.0	0.1	0.1	1.4	0.0
**Seed2**	2.9	1.1	1.3	0.1	25.7	0.3	0.0	0.0	0.1	0.4	0.1	0.0	15.4	0.0
**Embryo3**	3.3	26.2	47.5	3.7	0.7	0.0	3.4	0.1	23.3	0.1	0.3	0.2	2.6	4.5
**Embryo4**	0.3	2.3	55.6	0.7	5.3	0.0	4.2	0.1	23.8	0.0	0.6	0.4	0.7	1.2
**Embryo5**	0.5	0.1	38.6	0.4	3.5	0.0	4.3	0.8	15.8	0.0	4.4	0.4	2.7	2.2
**Ghost3**	5.4	38.2	6.2	5.2	6.4	0.0	0.0	0.0	0.1	1.7	0.8	0.0	55.2	2.3
**Ghost4**	0.6	44.6	10.1	25.3	4.6	0.0	0.0	0.0	0.4	2.9	0.4	0.0	113.5	13.7
**Ghost5**	2.9	21.6	6.2	96.3	0.9	0.0	0.1	0.0	1.4	31.4	0.8	0.0	318.9	73.2
**Wall1**	0.0	0.0	0.6	0.0	36.9	0.0	0.1	0.0	0.1	0.0	0.0	0.0	5.6	0.0
**Wall2**	0.4	0.0	0.3	0.0	17.5	0.1	0.0	0.0	0.0	0.1	0.0	0.0	11.7	0.0
**Wall3**	0.6	2.9	0.6	0.4	20.9	0.0	0.0	0.0	0.0	0.4	0.0	0.0	28.5	0.3
**Wall4**	0.1	6.2	2.0	2.6	12.8	0.0	0.0	0.0	0.3	0.6	0.0	0.0	46.6	0.7
**Wall5**	0.0	0.4	1.8	1.9	13.7	0.0	0.2	0.0	0.7	0.0	0.1	0.0	21.3	1.6
**Cortex1**	0.0	0.0	1.5	0.0	39.4	0.0	0.0	0.0	0.3	0.0	0.0	0.0	7.6	0.0
**Cortex2**	0.0	0.0	0.5	0.0	48.5	0.0	0.5	0.0	0.2	0.0	0.0	0.0	3.0	0.0
**Cortex3**	0.0	0.0	0.1	0.0	29.1	0.0	0.1	0.0	0.1	0.0	0.0	0.0	3.5	0.0
**Cortex4**	0.0	0.0	0.1	0.0	32.3	0.0	0.0	0.0	0.0	0.0	0.0	0.0	3.2	0.0
**Cortex5**	0.0	0.0	0.2	0.0	13.7	0.0	0.0	0.0	0.0	0.0	0.0	0.0	1.8	0.0
**Pith1**	0.0	0.0	1.7	0.0	25.9	0.0	0.0	0.0	0.6	0.0	0.0	0.0	4.1	0.0
**Pith2**	0.0	0.0	0.3	0.0	46.6	0.0	0.0	0.0	0.1	0.0	0.0	0.0	1.9	0.0
**Pith3**	0.0	0.0	0.2	0.0	29.3	0.0	0.0	0.0	0.3	0.0	0.0	0.0	1.9	0.0
**Pith4**	0.0	0.0	0.2	0.0	24.4	0.0	0.0	0.0	0.0	0.0	0.0	0.0	1.1	0.0
**Pith5**	0.0	0.0	0.6	0.0	13.7	0.0	0.0	0.0	0.0	0.0	0.0	0.0	0.5	0.0
**Carpel78**	0.0	0.0	6.0	0.0	31.9	0.0	0.6	0.0	4.4	0.0	0.1	0.0	3.2	0.0
**Carpel9**	0.0	0.0	6.6	0.0	32.5	0.0	0.3	0.0	3.5	0.0	0.0	0.0	2.2	0.0
**Carpel10**	0.0	0.0	5.1	0.0	52.9	0.0	1.3	0.0	2.3	0.0	0.9	0.0	5.5	0.0
**Carpel12**	0.0	0.0	1.8	0.0	53.5	0.0	1.3	0.2	0.3	0.0	1.3	0.0	5.3	0.0
**Anther78**	0.0	0.0	1.4	0.0	14.7	0.1	2.2	0.2	2.8	0.0	0.0	0.0	2.4	0.0
**Anther9**	0.2	0.0	3.3	0.0	13.5	0.0	0.4	0.0	1.6	0.0	1.2	0.0	4.8	0.1
**Anther10**	2.7	0.0	6.8	0.0	7.0	0.0	0.2	0.0	2.5	0.0	6.2	0.0	1.9	0.0
**Anther11**	16.2	0.0	5.0	0.0	3.1	0.0	0.6	0.0	0.1	0.0	11.6	0.0	2.1	0.0
**Anther12**	48.4	0.5	5.9	0.0	3.6	0.0	0.3	0.0	0.0	0.0	13.1	0.0	1.4	0.0
**Pollen**	10.8	2.2	8.3	0.0	0.8	0.0	0.0	0.0	0.0	0.0	2.7	0.0	0.0	16.4
**Style1**	0.0	0.0	26.8	0.0	20.3	0.0	1.8	2.5	0.1	0.0	1.0	0.0	3.0	0.0
**Style2**	0.1	0.2	6.1	0.0	88.3	0.0	0.4	0.2	0.0	0.0	0.1	0.0	4.3	2.5
**Leaf**	0.0	0.0	3.6	0.0	89.9	0.0	3.1	0.1	2.3	0.0	3.6	0.1	34.0	0.0
**Seedling**	0.0	0.0	2.1	0.0	13.4	0.0	0.1	5.2	0.1	0.0	1.2	0.0	4.5	0.0
**Root**	0.5	0.0	2.3	0.0	3.0	0.0	0.9	3.9	0.2	0.0	3.4	0.3	19.2	0.0
